# Thalamocortical Circuit Controls Neuropathic Pain *via* Up-regulation of HCN2 in the Ventral Posterolateral Thalamus

**DOI:** 10.1007/s12264-022-00989-5

**Published:** 2022-12-20

**Authors:** Yi Yan, Mengye Zhu, Xuezhong Cao, Gang Xu, Wei Shen, Fan Li, Jinjin Zhang, Lingyun Luo, Xuexue Zhang, Daying Zhang, Tao Liu

**Affiliations:** 1 Department of Pain Medicine, The First Affiliated Hospital of Nanchang University, Nanchang, 330006 China; 2 Institute of Pain Medicine, Jiangxi Academy of Clinical and Medical Sciences, Nanchang, 330006 China; 3 Key Laboratory of Neuropathic Pain, Healthcare Commission of Jiangxi Province, Nanchang, 330006 China; 4 Center for Experimental Medicine, the First Affiliated Hospital of Nanchang University, Nanchang, 330006 China

**Keywords:** Neuropathic pain, Thalamocortical circuit, HCN2 channel, Optogenetics, Electrophysiology

## Abstract

The thalamocortical (TC) circuit is closely associated with pain processing. The hyperpolarization-activated cyclic nucleotide-gated (HCN) 2 channel is predominantly expressed in the ventral posterolateral thalamus (VPL) that has been shown to mediate neuropathic pain. However, the role of VPL HCN2 in modulating TC circuit activity is largely unknown. Here, by using optogenetics, neuronal tracing, electrophysiological recordings, and virus knockdown strategies, we showed that the activation of VPL TC neurons potentiates excitatory synaptic transmission to the hindlimb region of the primary somatosensory cortex (S1HL) as well as mechanical hypersensitivity following spared nerve injury (SNI)-induced neuropathic pain in mice. Either pharmacological blockade or virus knockdown of HCN2 (shRNA-*Hcn2*) in the VPL was sufficient to alleviate SNI-induced hyperalgesia. Moreover, shRNA-*Hcn2* decreased the excitability of TC neurons and synaptic transmission of the VPL–S1HL circuit. Together, our studies provide a novel mechanism by which HCN2 enhances the excitability of the TC circuit to facilitate neuropathic pain.

**Supplementary Information:**

The online version contains supplementary material available at 10.1007/s12264-022-00989-5.

## Introduction

Neuropathic pain is caused by a lesion or disease affecting the somatosensory nervous system [1–3]. It is one of the most difficult-to-treat pain syndromes that affects 6.9% to 10% of the general population [2, 4, 5]. Chronic neuropathic pain dramatically impairs patients’ quality of life and has imposed an enormous economic burden on healthcare and social support systems [5, 6]. However, since the mechanisms underlying neuropathic pain have not yet been fully elucidated, first-line medications usually provide unsatisfactory outcomes, and neuropathic pain remains a tremendous clinical challenge [7–9]. For this reason, a better understanding of the comprehensive mechanisms underlying neuropathic pain may lead to novel therapeutic strategies [10, 11].

The ventral posterolateral thalamus (VPL) is one of the most critical parts of the somatosensory thalamic nuclei. It receives inputs from the peripheral nociceptors of the limbs and projects to the corresponding cortical sensory regions and the limbic system; it is called the thalamocortical (TC) circuit [12–14]. Therefore, VPL neurons are also referred to as TC neurons. Human imaging studies have revealed multiple ectopic changes in VPL responses to neuropathic pain, including abnormal volume and connectivity [15, 16]. In addition, the VPL is a common target for deep brain stimulation in the treatment of neuropathic pain [17–19]. Despite the fact that a number of studies have shown that the firing pattern of TC neurons is altered in neuropathic pain and a decrease in the ectopic firing reverses neuropathic pain-like behavior [20–22], how the TC neurons and TC circuit participate in the pathogenesis of neuropathic pain is largely uncharacterized.

Hyperpolarization-activated cyclic nucleotide-gated (HCN) channels are activated upon hyperpolarization, generating a mixed Na^+^/K^+^ inward cation current (*I*_h_) [11, 23]. They are widely expressed in the central nervous system and indispensable for regulating neuronal membrane potential, input resistance, and synaptic transmission [24–27]. Consequently, HCN channels have been linked to many neurological disorders, such as neuropathic pain, epilepsy, and psycho-affective disorders [23]. HCN2, one of the four HCN isoforms (HCN1–4), plays a dominant role in neuropathic pain [25, 28, 29]. For instance, specifically deleting HCN2 in dorsal root ganglion (DRG) neurons expressing Na_V_1.8 reverses hyperalgesia in the chronic constriction injury model [30]. In addition, HCN2 has also been shown to be up-regulated in the ventrolateral periaqueductal gray and thalamus after neuropathic pain, while local injection of ZD7288, a selective HCN channel blocker, attenuates both the mechanical allodynia and thermal hyperalgesia [31–33]. The increased expression of HCN2 in the thalamus under neuropathic pain may result in hyperexcitability of TC neurons, which might activate the TC circuit, although these need to be further validated. To address these questions, we applied a combination of behavioral, *in vitro* electrophysiological, optogenetics, pharmacological, and virus-knockdown techniques. We found that silencing the HCN2 channels in TC neurons decreases their excitability and ectopic synaptic transmission of the VPL-S1HL circuit and reverses SNI-induced hyperalgesia. Together, our findings unveil the unknown role of HCN2 in the TC circuit and nociceptive pain.

## Materials and Methods

### Animals

Adult C57BL/6J male mice (8–10 weeks old) were obtained from the Animal Center of Nanchang University. The mice were housed in a temperature-controlled room, maintained at 23 ± 2 °C and 40%–60% humidity, with a 12:12-h light/dark cycle. Food and water were available *ad libitum*. The experimental procedures were approved by the Institutional Animal Care and Use Committee of the First Affiliated Hospital of Nanchang University. The number of animals and procedures involving pain has been minimized in compliance with the guidelines of the 3Rs (Replacement, Reduction, and Refinement).

### SNI

We used the SNI model to establish neuropathic pain in mice as previously described [34]. Briefly, under isoflurane (2%) (RWD Life Science, Shenzhen, China) inhalation anesthesia, the fur of the left thigh was shaved and disinfected. After incising the skin and exposing the sciatic nerve trunk and its branches (the tibial, common peroneal, and sural nerves), the tibial and common peroneal nerves were ligated and cut, with the sural nerve remaining intact. The muscles and skin were then sutured. The sham procedures were the same as those in SNI, except for ligation and amputation of the tibial and common peroneal nerves.

### Behavioral Tests

#### Mechanical Allodynia

Mechanical allodynia was measured using an electronic von Frey aesthesiometer (BIO-EVF4 S, Bioseb, Vitrolles, France) [35]. Animals were placed in plastic cages with a wire-mesh floor. The von Frey filaments were applied to the lateral plantar surface of the hind paw with increasing force. The paw withdrawal threshold (PWT) was defined as the lowest pressure (g) that evoked a brisk withdrawal reflex of the paw. The PWT was measured at 0, 1, 3, 5, 7, 10, and 14 days after SNI. The PWT values were averaged over three tests at intervals of 1 min.

#### Open Field Test (OFT)

The OFT was used to evaluate the locomotor activity of mice after drug injection. In brief, mice were individually placed in the center of a box (50 cm × 50 cm × 25 cm) and allowed to explore freely for 30 min. The total distances moved were recorded by a video camera and analyzed using MatLab (MathWorks, MA, USA).

### Western Blot

After the behavioral test, fresh VPL tissue was homogenized in ice-cold radio immunoprecipitation assay lysis buffer and 1% protease inhibitor for 1 h and then centrifuged at 12,000 r/min for 15 min at 4 °C. For electrophoretic blotting, samples were separated by 8% sodium dodecyl sulfate-polyacrylamide gel electrophoresis and transferred to a nitrocellulose membrane. Subsequently, the membrane was blocked at room temperature for 1 h and incubated with rabbit polyclonal anti-HCN1 (1:1000; Proteintech, IL, USA) and anti-HCN2 (1:1000; Proteintech) or rabbit polyclonal anti-β-actin (1:1000; Proteintech) antibodies at 4 °C overnight. After 3 washes, the membranes were incubated with goat anti-rabbit IgG secondary antibody (1:2000; Thermo Fisher Scientific, MA, USA) for 2 h at room temperature. The immunoreactive bands were visualized by an iBright 1500 imaging system (Thermo Fisher Scientific) and quantified by using ImageJ software (http://rsb.info.nih.gov/ij/).

### Immunofluorescence Staining

The mice were anesthetized with urethane (1.5 g/kg), perfused with cold saline, and fixed with 4% paraformaldehyde (PFA). Subsequently, the brains were removed and fixed with 4% PFA for 6 h, then immersed in 30% sucrose for dehydration for at least 3 days. Next, the brains were cut into coronal sections at 30 μm on a freezing microtome (CM1950; Leica, Nussloch, Germany). The sections were washed 3 times with phosphate-buffered saline and then incubated in a blocking solution (2% donkey serum, 2% bovine serum albumin, and 0.3% Triton X-100) for 30 min. Next, the sections were incubated with mouse monoclonal anti-parvalbumin antibody (1:1000; Sigma-Aldrich, MO, USA) and/or guinea pig polyclonal anti-NeuN (1:200; Synaptic Systems, Göttingen, Germany) at 4 °C for 12 h. After washing, the sections were incubated with donkey anti-mouse Alexa Fluor® 647 or donkey anti-mouse Alexa Fluor® 546 (1:400; Thermo Fisher Scientific) or donkey anti-guinea pig Cyanine 5-labeled secondary antibody (1:400; Jackson ImmunoResearch, PA, USA) at 4 °C for 12 h. Finally, the sections were imaged on a confocal microscope (LSM700, Zeiss, Oberkochen, Germany).

### Cannula Implantation and Virus Injection

Mice were anesthetized with isoflurane and placed in a stereotaxic instrument (RWD Life Science). We targeted the VPL with stereotaxic coordinates ranging from bregma in mm [anterior-posterior (AP): − 1.70; medial-lateral (ML): − 1.70; dorsal-ventral (DV): − 3.80] according to the mouse brain atlas [36]. A guide cannula (RWD Life Science) was implanted in the VPL contralateral to the SNI. The guide cannula was fixed to the skull using dental acrylic and skull screws. All mice were allowed to recover for at least one week before further experiments. Saline or ZD7288 (Tocris, Bristol, UK) solutions (5 μg/kg and 10 μg/kg) were infused into the VPL using a microinjection unit that extended 0.5 mm beyond the tip of the guide cannula. The microinjection unit was attached to a Hamilton microsyringe *via* polyethylene tubing. Saline and drug solutions were infused into the VPL (300 nL, 100 nL/min). The microinjection unit was held for 10 min before withdrawal. ZD7288 was dissolved in sterile saline.

For virus transduction, using a Hamilton microsyringe, mice were injected with 150 nL (10 nL/min) virus expressing pAAV-*CaMKIIα-hChR2(H134R)-mCherry*-*WPRE* (AAV-*CaMKII-ChR2-mCherry*) or pAAV-*CaMKIIα-NpHR3.0-mCherry-WPRE* (AAV*-CaMKII-NpHR-mCherry*) (Obio Technology, Shanghai, China) and pAAV-*U6*-shRNA-scramble-*CMV-eGFP-3Flag* (AAV-shRNA-scramble-*GFP*) or pAAV-*U6*-shRNA-*Hcn2*-*CMV-eGFP-3Flag* (AAV-shRNA-*Hcn2-GFP*) (Genechem, Shanghai, China) into the VPL contralateral to the SNI. After completion of the infusion, the needle was left in place for 10 min before withdrawal to ensure adequate diffusion. All mice were allowed to recover for at least three weeks before further experiments.

For retrograde tracing, 1% Fluoro-Gold (FG) (Biotium, CA, USA) (40 nL, 10 nL/min) was injected into the S1HL with stereotaxic coordinates ranging from bregma in mm (AP: − 0.95; ML: − 1.50; DV: − 0.80). Brain sections were prepared for tracing the FG signal one week later.

### Acute Brain Slice Preparation

Brain slices were prepared as previously described [37]. Briefly, urethane (1.5 g/kg, i.p.)-anesthetized mice were transcardially perfused with ice-cold sucrose-based artificial cerebrospinal fluid (sucrose-ACSF) that contained (in mmol/L): 204 sucrose, 2.5 KCl, 3.5 MgCl_2_, 0.5 CaCl_2_, 1.25 NaH_2_PO_4_, 2 sodium pyruvate, 11 D-glucose, 25 NaHCO_3_, and 1 kynurenic acid. Then the brain was mounted on a vibratome (VT1000S, Leica) cutting stage in the same solution, and coronal slices (280 μm) containing the S1HL or the VPL were cut. Sucrose-ACSF was bubbled with 95% O_2_ and 5% CO_2_ for at least 10 min before use. The brain slices were incubated with ACSF that contained (in mmol/L) 117 NaCl, 3.6 KCl, 2.5 CaCl_2_, 1.2 MgCl_2_, 1.2 NaH_2_PO_4_, 25 NaHCO_3_, 11 D-glucose, and 2 sodium pyruvate, and gassed with 95% O_2_ and 5% CO_2_ at 32 °C and then at room temperature for 30 min.

### *In Vitro* Electrophysiology

After recovery, the brain slices were transferred into a recording chamber and constantly perfused with standard ACSF at room temperature (23–25 °C) at a perfusion rate of 2–4 mL/min. Neurons in layer IV (L4) of the S1HL and VPL were visualized under an Olympus microscope (BX51WI, Olympus, Tokyo, Japan). Whole-cell patch-clamp recordings were obtained from visually identified neurons. GFP or mCherry-positive neurons were identified using a 40× objective (Olympus) with blue (470 nm) or yellow light (590 nm) (M470L4-C1 or M590L4-C1, Thorlabs Inc., NJ, USA) and neurobiotin (Vector Laboratories, CA, USA) was loaded in a subset of these neurons during recording. Unless otherwise noted, patch pipettes (3–6 MΩ) were pulled from borosilicate glass capillaries (World Precision Instruments, FL, USA) with a Sutter P-97 puller (Sutter Instruments, CA, USA) and were filled with an intrapipette solution that contained (in mmol/L): 130 K-gluconate, 5 KCl, 10 Na_2_-phosphocreatine, 0.5 EGTA, 10 HEPES, 4 Mg-ATP, and 0.3 Li-GTP (pH 7.3–7.4 adjusted with KOH; osmolarity = 285–295 mOsm). All signals were recorded *via* an EPC-10 amplifier and Patchmaster software (HEKA Electronics, Lambrecht, Germany) and analyzed with Clampfit 10.7 software (Molecular Devices, CA, USA) or the MiniAnalysis program (Synaptosoft Inc., Decatur, GA, USA). The series resistance was kept at 10–30 MΩ, and neurons with series resistance changes >20% were excluded from the analysis.

The resting membrane potential (RMP, in mV) of TC neurons was recorded within 20 s after establishing the whole-cell configuration, with no current applied (*I* = 0 pA). Input resistance (in MΩ) was measured from the current response to a 10-mV hyperpolarizing step in voltage clamp. Rheobase current (in pA) was determined as the smallest current step to elicit at least one action potential (AP). The spike threshold (in mV) was defined as the inflection point during the spike initiation of the first AP. Spike amplitude (in mV) was defined as the difference between the firing threshold and its maximum positive peak. Spike half-width (in ms) was calculated at 50% of spike amplitude. In voltage-clamp recording, *I*_h_ was recorded *via* voltage-clamp at − 40 mV, and a series of hyperpolarizing voltage steps (from − 50 mV to − 130 mV in 10-mV decrements) with 1 μmol/L tetrodotoxin (TTX) (Tocris), 1 mmol/L BaCl_2_ (Sigma, MA, USA), 1 mmol/L 4-aminopyridine (4-AP) (Tocris), and 0.1 mmol/L NiCl_2_ (Sigma) in the ACSF [37].

Optical stimulation was delivered by a collimated light-emitting diode (LED) with 470 or 590 nm peak wavelength (Thorlabs Inc.) with a stimulator Master 8 (AMP Instruments Ltd., Jerusalem, Israel) and delivered to the brain slice through a 40× objective (Olympus). To test the function of AAV-*CaMKII-ChR2-mCherry*, TC neurons expressing ChR2 were stimulated with blue light (470 nm, 10 μW, 1 ms) using 5 Hz, 10 Hz, and 20 Hz stimulation protocols. Similarly, TC neurons expressing NpHR(*Natronomonas pharaonis* halorhodopsin) were stimulated with yellow light (590 nm, 10 μW, 500 ms) at 0 pA and with a depolarizing current pulse (100 pA, 1 s).

To record excitatory postsynaptic currents (EPSCs), S1HL neurons were voltage-clamped at − 70 mV. TTX (1 μmol/L) was included in the ACSF for miniature EPSC (mEPSC) recording. To record optogenetically-evoked EPSCs (oEPSCs), blue light (470 nm, 5 μW, 1 ms) was delivered to the S1HL in mice in which the VPL had been virally transduced with AAV-*CaMKII-ChR2-mCherry*. The optically-stimulated paired-pulse ratio (PPR) was calculated with paired-pulse stimuli of blue light (470 nm, 5 μW, 1 ms) at a 200-ms interval; 5 μW was chosen as the optimum light intensity, as we found that the VPL outputs were so strong that sometimes spikes were elicited in S1HL neurons voltage-clamped at − 70 mV. Monosynaptic oEPSCs were identified by using TTX (1 μmol/L), 4-AP (1 mmol/L), and 6-cyano-7-nitroquinoxaline-2,3-dione (CNQX, 10 μmol/L). To assess the effect of VPL neurons projecting to the S1HL, current-clamp recording (*I* = 0 pA) was carried out on S1HL L4 neurons with blue light stimulation (470 nm, 10 μW, 1 ms, 10 Hz) to the axon terminals of TC neurons in the S1HL. All the reagents used for the electrophysiological experiment were from Sigma-Aldrich unless otherwise noted.

### Optogenetic Stimulation *In Vivo*

Two weeks after virus injection, optical fibers (200 μm in diameter; Newdoon, Hangzhou, China) were implanted into the VPL (AP: − 1.70 mm; ML: − 1.70 mm; DV: − 3.60 mm) and the S1HL (AP: − 0.95 mm; ML: − 1.50 mm; DV: − 0.60 mm) in a stereotaxic apparatus under anesthesia. The fiber was fixed to the skull using dental acrylic and skull screws. After one week, each fiber was connected *via* optical fiber sleeves to a laser generator and controller (Aurora-400, Newdoon) with stimulation protocols of a 15-min pulse of blue light (470 nm, 10 mW, 1-ms pulses, 20 Hz) or yellow light (589 nm, 10 mW, 1-ms pulses, 20 Hz). The location of the fibers was determined after all experiments.

### Statistical Analyses

All data were analyzed and expressed with GraphPad Prism 8 (GraphPad Software, CA, USA). All numerical values are presented as the mean ± SEM. The Shapiro-Wilk test was used to assess the normal distribution of data. The Levene test was used to test the homogeneity of variance. The Mann-Whitney *U* test or *t*-test was used as appropriate for the comparison of two groups. One-way or two-way analysis of variance (ANOVA) followed by the Bonferroni *post hoc* test was used to compare multiple groups. Significance was set at *P* <0.05.

## Results

### Excitability of TC Neurons in the VPL Increases in Mice with Nerve Injury

**Fig. 1:**
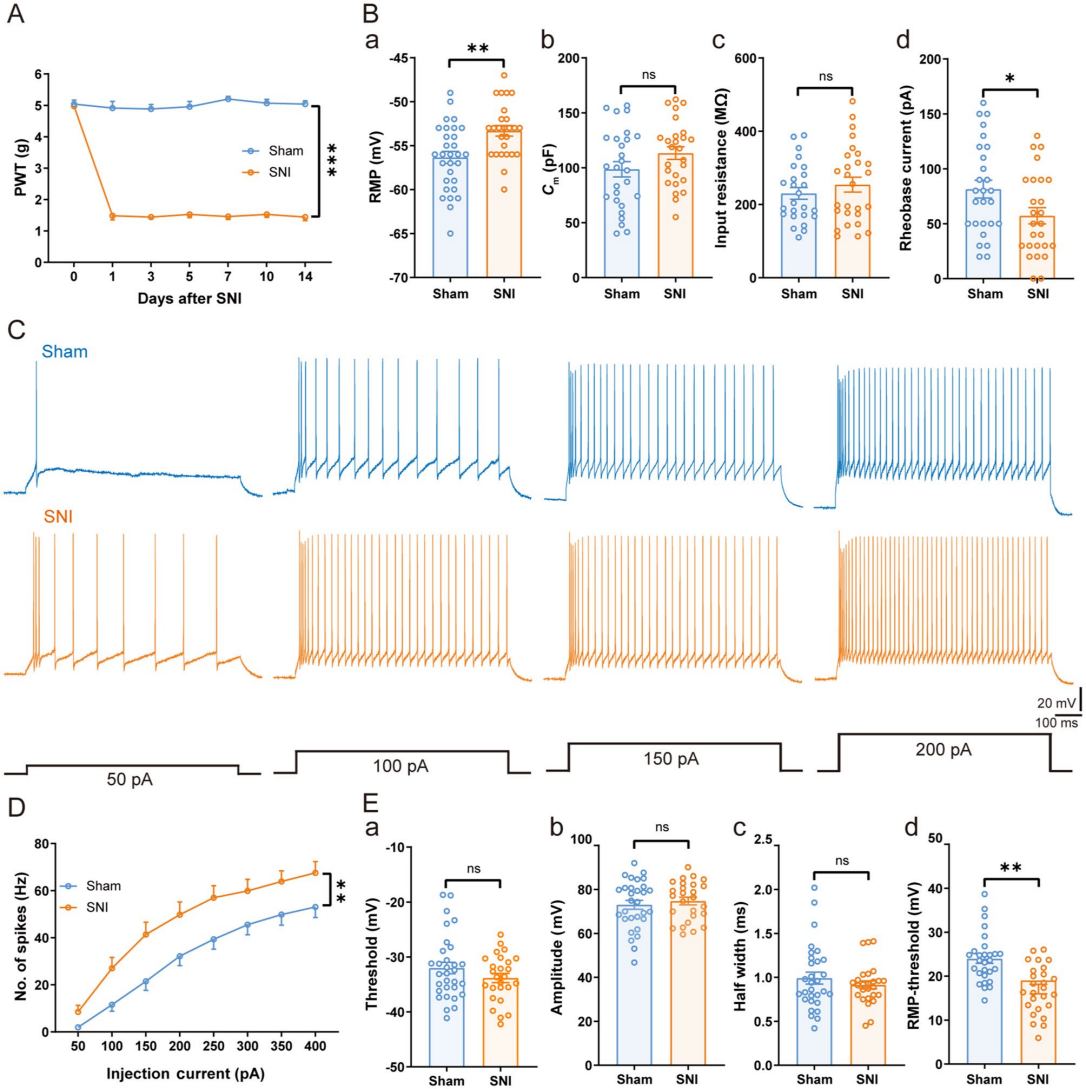
Increased excitability of VPL TC neurons under neuropathic pain states. **A** Time courses of SNI-induced changes in mechanical pain thresholds to von Frey filaments (*n* = 7 mice per group, two-way ANOVA). **B** Summary of the passive membrane properties of VPL TC neurons in sham and SNI mice after 2 weeks. **Ba** RMP; **Bb** *C*_m_; **Bc** input resistance; **Bd** rheobase current (*n* = 27–31 cells from 11 sham mice, *n* = 26 cells from 13 SNI mice, *t-*test). **C** Representative traces of depolarizing current-elicited spikes recorded from VPL TC neurons in sham and SNI mice. **D** Frequency-current (*F*-*I*) curves showing the number of spikes in VPL TC neuronal responses to a series of 1-s current pulses from 50 pA to 400 pA in 50-pA steps (*n* = 25 cells from 10 sham mice, *n* = 23 cells from 11 SNI mice, two-way ANOVA). **E** Summary of the active membrane properties of VPL TC neurons in sham and SNI mice. **Ea** Spike threshold. **Eb** Spike amplitude. **Ec** Spike half-width. **Ed** RMP-spike threshold (*n* = 27–31 cells from 11 sham mice, *n* = 26 cells from 13 SNI mice, *t-*test). For **A, B, D**, and **E**: *P <0.05, **P <0.01, ***P <0.001; ns, no significant difference. VPL, ventral posterolateral thalamus; TC, thalamocortical; SNI, spared nerve injury; PWT, paw withdrawal threshold; RMP, resting membrane potential; *C*_m_, membrane capacitance

To investigate the role of VPL TC neurons in neuropathic pain, we used a model of peripheral neuropathy (SNI). After SNI, mice developed neuropathic pain-like behavior, assessed as mechanical allodynia that started on day 1 and persisted for at least 14 days after nerve injury [*F*_(1, 6)_ = 727.80, *P* <0.001] (Fig. 1A). To address whether this resulted from hyperexcitation in the VPL, brain slices from sham and SNI mice (at 14 days after injury) were prepared for whole-cell patch-clamp recordings in VPL TC neurons. Compared with mice after sham surgery, the RMP was more depolarized in SNI mice [SNI: − 53.31 ± 0.60 mV *vs* sham: − 56.35 ± 0.66 mV, *t*_(55)_ = 3.34, *P* <0.01] (Fig. 1Ba). There was no difference between the two groups in the membrane capacitance (*C*_m_) [SNI: 113.40 ± 5.77 pF *vs* sham: 98.63 ± 6.97 pF, *t*_(51)_ = 1.62, *P* = 0.11] (Fig. 1Bb) and input resistance [SNI: 254.30 ± 20.46 MΩ *vs* sham: 230.30 ± 16.11 MΩ, *t*_(50)_ = 0.91, *P* = 0.37] (Fig. 1Bc). Moreover, the rheobase current was significantly smaller in the SNI group than in sham controls [SNI: 57.31 ± 7.41 pA *vs* sham: 81.48 ± 8.05 pA, *t*_(51)_ = 2.20, *P* <0.05] (Fig. 1Bd), suggesting increased excitability of VPL TC neurons after SNI. Simultaneously, we found that the number of depolarizing current-elicited spikes [*F*_(1, 24)_ = 8.50, *P* <0.01] (Fig. 1C, D) was greater in SNI mice than in sham mice. There was no difference between the two groups in spike threshold [SNI: − 32.01 ± 1.05 mV *vs* sham: − 33.83 ± 0.84 mV, *t*_(55)_ = 1.32, *P* = 0.19] (Fig. 1Ea), spike amplitude [SNI: 74.73 ± 1.76 mV *vs* sham: 73.25 ± 2.09 mV, *t*_(53)_ = 0.53, *P* = 0.59] (Fig. 1Eb), and spike half-width [SNI: 0.91 ± 0.05 ms *vs* sham: 0.90 ± 0.05 ms, *t*_(51)_ = 0.12, *P* = 0.90] (Fig. 1Ec). However, TC neurons from SNI mice displayed a smaller potential difference between the spike threshold and RMP [SNI: 19.06 ± 1.08 mV *vs* sham: 23.97 ± 1.06 mV, *t*_(52)_ = 3.24, *P* <0.01] (Fig. 1Ed). These electrophysiological results demonstrated that TC neuronal excitability was elevated in nerve-injured mice.

### Optogenetic Manipulation of VPL TC Neurons Modulates Mechanical Allodynia

**Fig. 2:**
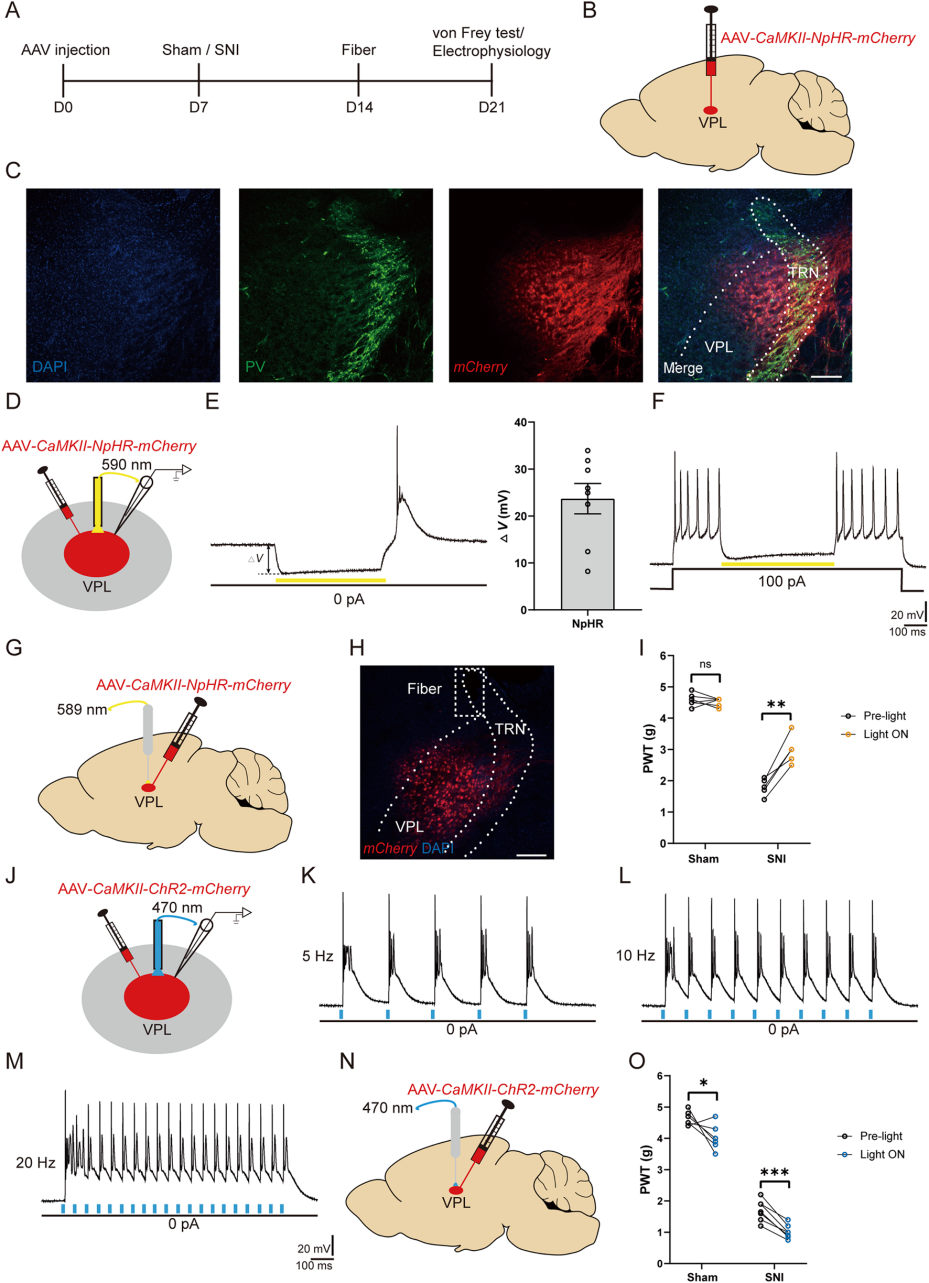
Optogenetic manipulation of VPL TC neurons modulates neuropathic pain behavior. **A** Timeline of experimental procedures. **B** Schematic of AAV-*CaMKII-NpHR-mCherry* injection into the VPL. **C** Representative confocal images confirming AAV-*CaMKII-NpHR-mCherry* (red) expression at the injection site in the VPL. PV neurons (green) are densely present in the TRN, which is closely adjacent to the VPL. Scale bar, 200 μm. **D** Schematic of AAV-*CaMKII-NpHR-mCherry* injection in mice and whole-cell recording in brain slices. **E** Representative trace and quantification of the change in membrane potential (*I*_hold_ = 0 pA) recorded from AAV-*CaMKII-NpHR-mCherry*-positive TC neurons stimulated by 0.5-s yellow light (590 nm) (*n* = 8 cells from 2 mice). **F** Representative trace of depolarizing current-elicited spikes in AAV-*CaMKII-NpHR-mCherry*-positive TC neurons stimulated by 0.5-s yellow light (590 nm). **G** Schematic of the AAV-*CaMKII-NpHR-mCherry* injection into the VPL and fiber implantation. **H** Representative image confirming mCherry (red) expression at the injection site in the VPL and the location of the fiber. Scale bar, 200 μm. **I** Mechanical PWT in response to optogenetic inhibition of TC neurons in the VPL of sham (*n* = 6) and SNI (*n* = 5) mice (*t-*test). **J** Schematic of AAV-*CaMKII-ChR2-mCherry* injection in mice and whole-cell recording in brain slices. **K–M** Representative traces of current-clamp recording (*I*_hold_ = 0 pA) from AAV-*CaMKII-ChR2-mCherry*-positive TC neurons stimulated by 470 nm blue light at different frequencies (**K**, 5 Hz; **L**, 10 Hz; **M**, 20 Hz). **N** Schematic of AAV-*CaMKII-ChR2-mCherry* injection into the VPL and fiber implantation. **O** Mechanical PWT in response to optogenetic activation of TC neurons in the VPL of sham (*n* = 6) and SNI (*n* = 7) mice (*t-*test). For **I** and **O**: *P <0.05, **P <0.01, ***P <0.001; ns, no significant difference. SNI, spared nerve injury; VPL, ventral posterolateral thalamus; TRN, thalamic reticular nucleus; PV, parvalbumin; TC, thalamocortical; PWT, paw withdrawal threshold; D0, day 0

To test whether increased TC neuronal activity is required for mechanical hypersensitivity, we applied optogenetic tools to broadly manipulate the activity of TC neurons in the VPL. As pioneering studies have demonstrated that TC neurons in the VPL are mainly glutamatergic [14, 38], we first used NpHR to silence VPL neurons *via Camk2a* promoter-driven expression using recombinant adeno-associated vectors (AAV-*CaMKII-NpHR-mCherry*) (Fig. 2A, B). The thalamic reticular nucleus (TRN) has been demonstrated to be mainly composed of gamma-aminobutyric acid neurons identified by parvalbumin (PV) [39]. Our *post hoc* histological analysis revealed a considerable mCherry signal within the VPL (Fig. 2C), which is adjacent to the TRN. The efficacy of manipulating neuronal activity was also ascertained *via in vitro* patch-clamp whole-cell recordings from VPL TC neurons from these mice. Yellow light (590 nm) stimulation of NpHR (Fig. 2D) resulted in hyperpolarization of the neuronal membrane potential (23.69 ± 3.22 mV) under current-clamp and resting conditions (*I* = 0 pA) (Fig. 2E), and the suppression of depolarizing current-elicited spikes (Fig. 2F). In parallel, optogenetic inhibition of VPL TC neurons *in vivo* elevated the pain threshold from 1.78 ± 0.12 g to 2.98 ± 0.20 g in SNI mice [*t*_(4)_ = 6.71, *P* <0.01], without any effect on sham mice [Pre-light: 4.58 ± 0.08 g *vs* Light ON: 4.51 ± 0.05 g, *t*_(5)_ = 0.69, *P* = 0.52] (Fig. 2G, I). Next, TC neurons were optogenetically stimulated with blue light (470 nm) after the expression of AAV-*CaMKII-ChR2-mCherry* in the VPL (Fig. 2J). As a result, we found that ChR2 activation reliably elicited spikes at distinct frequencies (Fig. 2K–M). In contrast to the effects of VPL inhibition on pain, activation of VPL TC neurons *in vivo* strongly worsened the mechanical allodynia in both SNI [Pre-light: 1.69 ± 0.13 g *vs* Light ON: 1.01 ± 0.08 g, *t*_(6)_ = 7.68, *P* <0.001] and sham mice [Pre-light: 4.66 ± 0.09 g *vs* Light ON: 4.03 ± 0.17 g, *t*_(5)_ = 3.12, *P* <0.05] (Fig. 2N, O). Taken together, our results demonstrate that the activation of TC neurons is necessary and sufficient for SNI-induced mechanical allodynia.

### Excitatory Synaptic Transmission Is Increased in the VPL–S1HL Circuit of SNI Mice

**Fig. 3:**
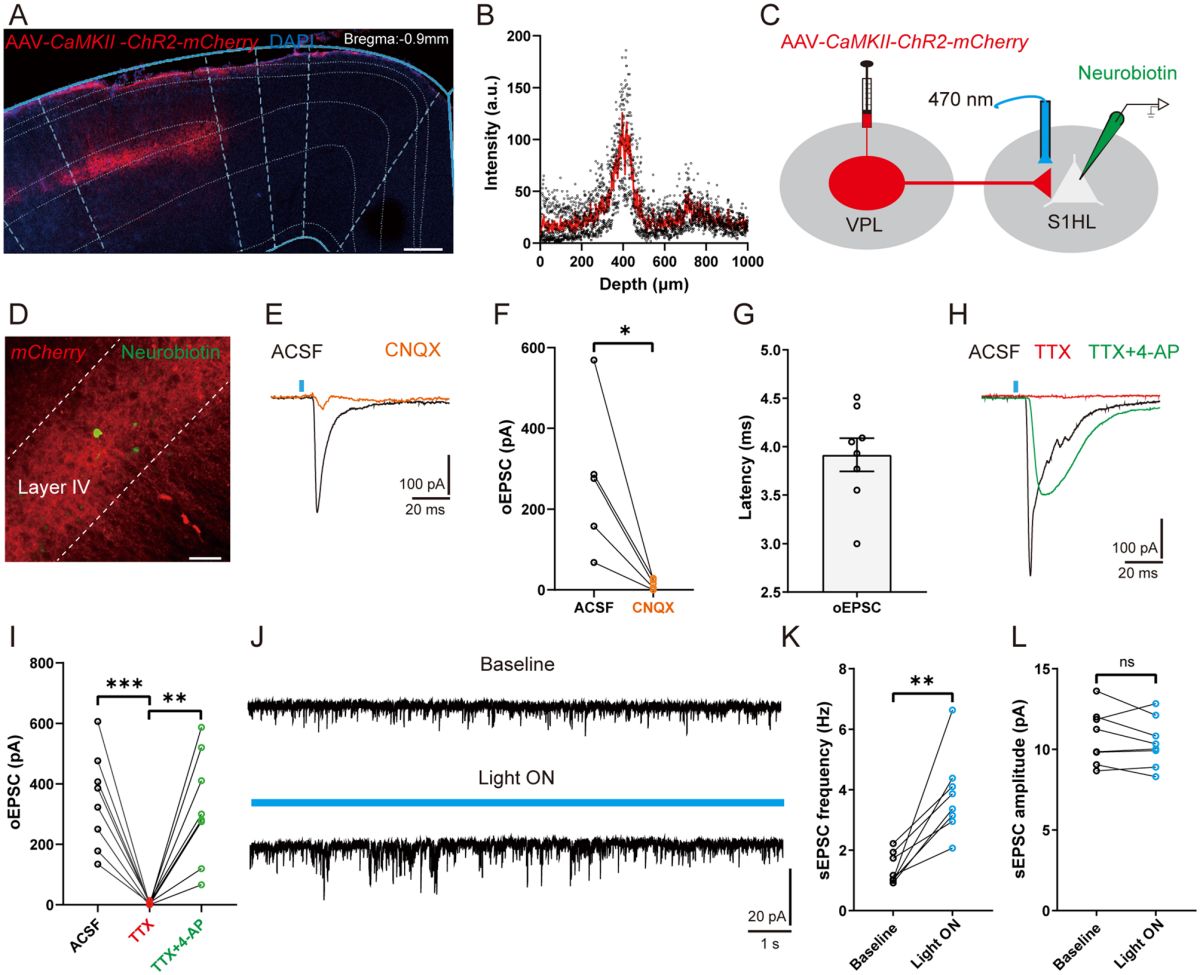
A TC circuit from VPL to S1HL. **A** Representative confocal image showing the expression of AAV-*CaMKII-ChR2-mCherry* in the S1HL (axon terminals). Scale bar, 200 μm. **B** Depth distributions of the fluorescent density of AAV-*CaMKII-ChR2-mCherry*-positive axon terminals in the S1HL (*n* = 3 mice). **C, D** Schematic (**C**) and representative confocal image (**D**) of AAV-*CaMKII-ChR2-mCherry-*positive axon terminals in the S1HL and whole-cell recording in RS neurons from S1HL slices. Neurobiotin was used to indicate recorded neurons. Scale bar, 50 μm. **E, F** Representative traces (**E**) and quantification (**F**) of oEPSCs in S1HL L4 RS neurons evoked by optogenetic activation of TC neuronal axon terminals in the presence of ACSF or the AMPA receptor antagonist CNQX (*n* = 5 cells from 3 mice, *t-*test). **G** Statistical data showing the oEPSC latency in S1HL L4 RS neurons (*n* = 8 cells from 4 mice). **H, I** Representative traces (**H**) and statistical data (**I**) of oEPSCs in the presence of ACSF, TTX, or TTX + 4-AP (*n* = 8 cells from 4 mice, *t-*test). **J** Representative traces of spontaneous EPSCs in S1HL L4 RS neurons at baseline and light ON with blue light (470 nm). **K, L** Statistical data showing the spontaneous EPSC frequency (**K**) and amplitude (**L**) of S1HL L4 RS neurons in the absence or presence of 470 nm light stimulation (*n* = 8 cells from 4 mice, *t-*test). For **F, G, I, K,** and **L**: *P <0.05, **P <0.01, ***P <0.001; ns, no significant difference. VPL, ventral posterolateral thalamus; S1HL, hindlimb region of the primary somatosensory cortex; RS, regular spiking; TC, thalamocortical; oEPSC, optogenetic excitatory postsynaptic current; L4, layer IV; AMPA, α-amino-3-hydroxy-5-methyl-4-isoxazolepropionic acid; CNQX, 6-cyano-7-nitroquinoxaline-2,3-dione

As the S1HL receives strong spinothalamic nociceptive input from the VPL [14, 22], we next interrogated whether the function of this TC circuit is changed under neuropathic pain conditions. To verify the existence of direct synaptic outputs from VPL to S1HL, we infused the AAV-*CaMKII-ChR2-mCherry* virus into the VPL. As shown in Fig. 3A, mCherry-positive axon terminals were robustly expressed in the S1HL, especially in L4, with a peak density at ~400 μm depth (Fig. 3B). Meanwhile, one week after microinjection of the retrograde tracer FG into the S1HL, FG-labeled cell bodies were observed in the VPL (Fig. S1), demonstrating an anatomical connection between VPL and S1HL. To further identify the functional connection, we made whole-cell recordings from excitatory neurons in L4, which displayed a regular spiking (RS) pattern and were clearly differentiated from inhibitory neurons that displayed fast spiking (Fig. S2). Optical stimulation of the mCherry-positive terminals of TC neurons in the S1HL elicited an oEPSC (latency: 3.71 ± 0.15 ms; amplitude: 271.40 ± 84.63 pA), which was abolished by the α-amino-3-hydroxy-5-methyl-4-isoxazolepropionic acid (AMPA) receptor antagonist CNQX [ACSF: 271.40 ± 84.63 pA *vs* CNQX: 15.00 ± 5.47 pA, *t*_(4)_ = 3.19, *P* <0.05] (Fig. 3C–G). We also found that TTX blocked the oEPSC [ACSF: 344.80 ± 55.53 pA *vs* TTX: 3.71 ± 1.74 pA, *t*_(7)_ = 6.10, *P* <0.001], which was restored by 4-AP [TTX: 3.71 ± 1.74 pA *vs* TTX+4-AP: 319.60 ± 63.83 pA, *t*_(7)_ = 4.92, *P* <0.01] (Fig. 3H, I). In addition, optogenetic activation of VPL TC neuronal axon terminals increased the frequency of the spontaneous EPSCs of RS neurons compared to baseline [baseline: 1.37 ± 0.17 Hz *vs* Light ON: 3.81 ± 0.48 Hz, *t*_(7)_ = 4.64, *P* <0.01], but not the amplitude [baseline: 10.77 ± 0.60 pA *vs* Light ON: 10.41 ± 0.54 pA, *t*_(7)_ = 1.19, *P* = 0.27] (Fig. 3J–L). These results demonstrate an excitatory monosynaptic circuit from VPL TC neurons to S1HL L4 RS neurons.

**Fig. 4:**
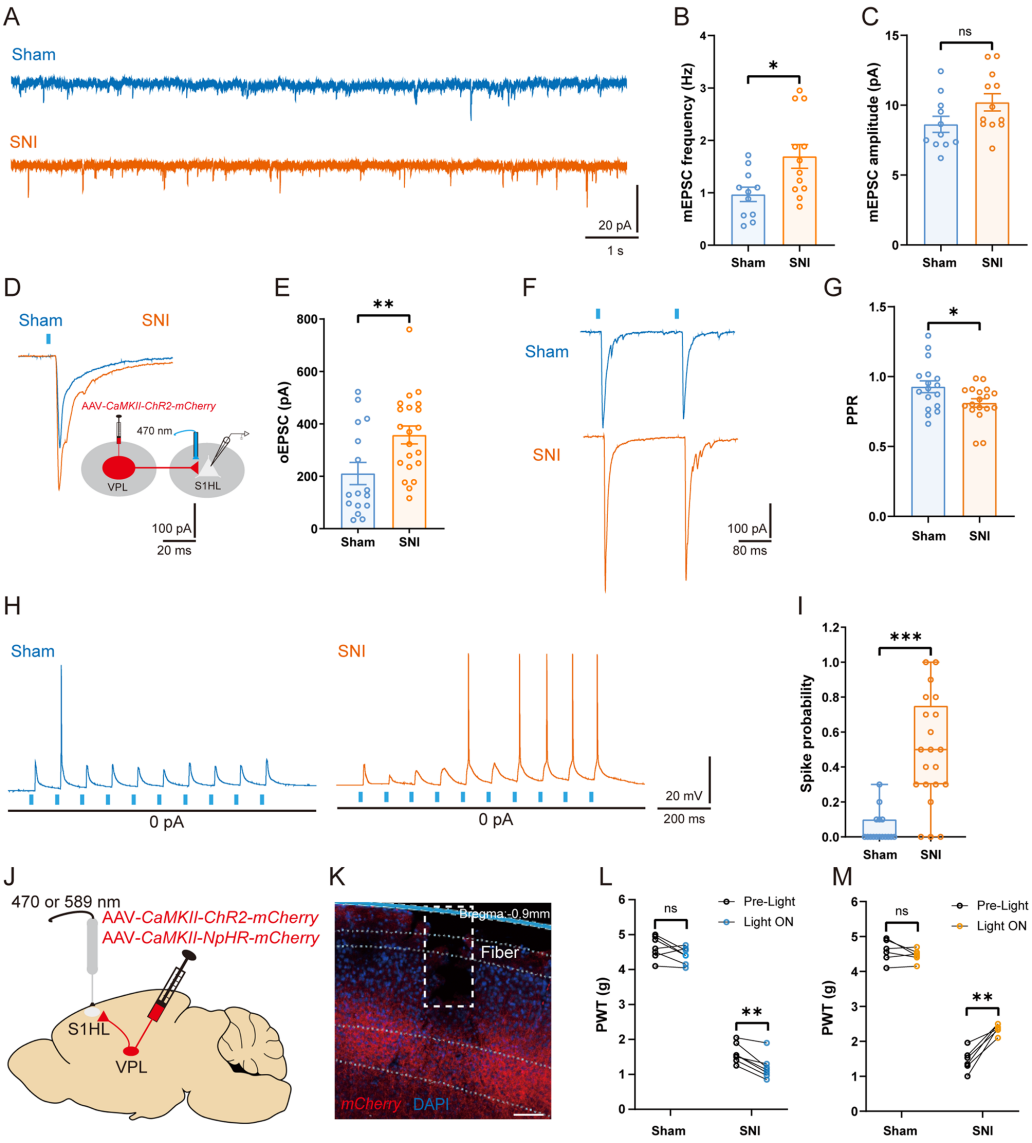
SNI increases the excitatory synaptic connections of the VPL–S1HL circuit. **A** Representative traces of mEPSCs recorded from S1HL L4 RS neurons at a holding potential of − 70 mV from sham and SNI mice. **B, C** Statistical data showing the mEPSC frequency (**B**) and amplitude (**C**) in S1HL L4 RS neurons from sham and SNI mice (*n* = 11 cells from 5 sham mice, *n* = 12 cells from 7 SNI mice, *t-*test). **D, E** Representative traces (**D**) and statistical data (**E**) of oEPSCs in S1HL L4 RS neurons from sham and SNI mice (*n* = 17 cells from 8 sham mice, *n* = 21 cells from 8 SNI mice, *t-*test). **F, G** Representative traces (**F**) and statistical data (**G**) of PPR in S1HL L4 RS neurons from sham and SNI mice (*n* = 17 cells from 8 sham mice, *n* = 18 cells from 8 SNI mice, *t-*test). **H, I** Representative traces (**H**) and statistical data (**I**) of spikes elicited by optogenetic stimulation of VPL outputs in S1HL L4 RS neurons from sham and SNI mice (*n* = 16 cells from 8 sham mice, *n* = 21 cells from 8 SNI mice, Mann-Whitney *U* test). **J** Schematic of AAV-*CaMKII-NpHR-mCherry* or AAV-*CaMKII-ChR2-mCherry* injection into the VPL and fiber implantation in the S1HL. **K** Representative confocal image confirming the mCherry-positive axon terminals of TC neurons in the S1HL and the location of a fiber. Scale bar, 100 μm. **L** Effects of optogenetic activation of mCherry-positive axon terminals of TC neurons in the S1HL on PWT in sham and SNI mice (*n* = 7 per group, *t-*test). **M** Effects of optogenetic inhibition of mCherry-positive axon terminals of TC neurons in the S1HL on PWT in sham and SNI mice (*n* = 6 per group, *t-*test). *P <0.05, **P <0.01, ***P <0.001 for **B, C, E, G, I, L**, and **M**. SNI, spared nerve injury; VPL, ventral posterolateral thalamus; S1HL, hindlimb region of the primary somatosensory cortex; RS, regular spiking; TC, thalamocortical; PPR, paired-pulse ratio; PWT, paw withdrawal threshold; mEPSC, miniature excitatory postsynaptic current; oEPSC, optogenetic excitatory postsynaptic current

Although SNI increased the excitability of VPL TC neurons, we wondered whether this would trigger enhanced excitatory synaptic input to L4 RS neurons of the S1HL. To test our hypothesis, we first compared the mEPSCs of L4 RS neurons from the contralateral S1HL (Fig. 4A). Compared to sham mice, the frequency of mEPSCs was greatly increased in SNI mice [SNI: 1.69 ± 0.23 Hz *vs* sham: 0.97 ± 0.14 Hz, *t*_(21)_ = 2.68, *P* <0.05] (Fig. 4B), but not the amplitude [SNI: 10.20 ± 0.61 pA *vs* sham: 8.63 ± 0.57 pA, *t*_(21)_ = 1.86, *P* = 0.08] (Fig. 4C), suggesting a presynaptic augmentation of transmitter release. Moreover, we found an increase in the number of depolarized current-elicited spikes [*F*_(1, 28)_ = 5.51, *P* <0.05] and a decrease in the rheobase current (SNI: 96.00 ± 12.88 pA *vs* sham: 130.60 ± 10.02 pA, *P* <0.05, Mann-Whitney test) in S1HL L4 RS neurons from SNI mice (Fig. S3A–C). To determine whether this increase is brought about by the activity of VPL TC neurons, the amplitudes of VPL-driven oEPSCs in RS neurons were analyzed. The results showed that the oEPSC amplitude of RS neurons increased in SNI mice following optogenetic stimulation of VPL axon terminals [SNI: 357.60 ± 34.03 pA *vs* sham: 210.60 ± 42.30 pA, *t*_(35)_ = 2.74, *P* <0.01] (Fig. 4D, E). Consistent with this, the optically-stimulated PPR was reduced in both sham and SNI groups, and it was lower in SNI mice than in the sham group [SNI: 0.81 ± 0.03 *vs* sham: 0.93 ± 0.04, *t*_(33)_ = 2.26, *P* <0.05] (Fig. 4F, G). Next, to unravel whether the enhanced synaptic transmission could affect the activity of postsynaptic neurons, VPL axon terminals that target the RS neurons in L4 of the S1HL were optically stimulated (Fig. 4H). Importantly, the spike probability was markedly higher in SNI than in the sham group (SNI: 0.48 ± 0.07 *vs* sham: 0.05 ± 0.02, *P* <0.001, Mann-Whitney test) (Fig. 4I). Taken together, these findings demonstrated that SNI potentiates excitatory synaptic inputs from the VPL to the S1HL *via* a presynaptic mechanism.

To assess the effect of *in vivo* manipulation of VPL outputs to the S1HL on the nociceptive behavioral response, we optogenetically activated or inhibited the axon terminals of VPL TC neurons in the S1HL (Fig. 4J, K). As shown in Fig. 4L, optical activation of the VPL-to-S1HL circuit decreased the PWT in SNI [Pre-light: 1.60 ± 0.11 g *vs* Light ON: 1.21 ± 0.13 g, *t*_(6)_ = 4.98, *P* <0.01], but not in sham mice [Pre-light: 4.63 ± 0.12 g *vs* Light ON: 4.40 ± 0.09 g, *t*_(6)_ = 1.95, *P* = 0.10]. In contrast, optical inhibition of this circuit significantly increased the PWT in the SNI group [Pre-light: 1.46 ± 0.13 g *vs* Light ON: 2.36 ± 0.06 g, *t*_(5)_ = 6.45, *P* <0.01], with no effect on sham mice [Pre-light: 4.59 ± 0.13 g *vs* Light ON: 4.46 ± 0.07 g, *t*_(5)_ = 1.33, *P* = 0.24] (Fig. 4M). These results demonstrate that the VPL–S1HL circuit brings about the sensitization of nociceptive behaviors *via* enhanced excitatory neurotransmission.

### Expression of HCN2 and *I*_h_ Amplitude Increase in SNI Mice

**Fig. 5:**
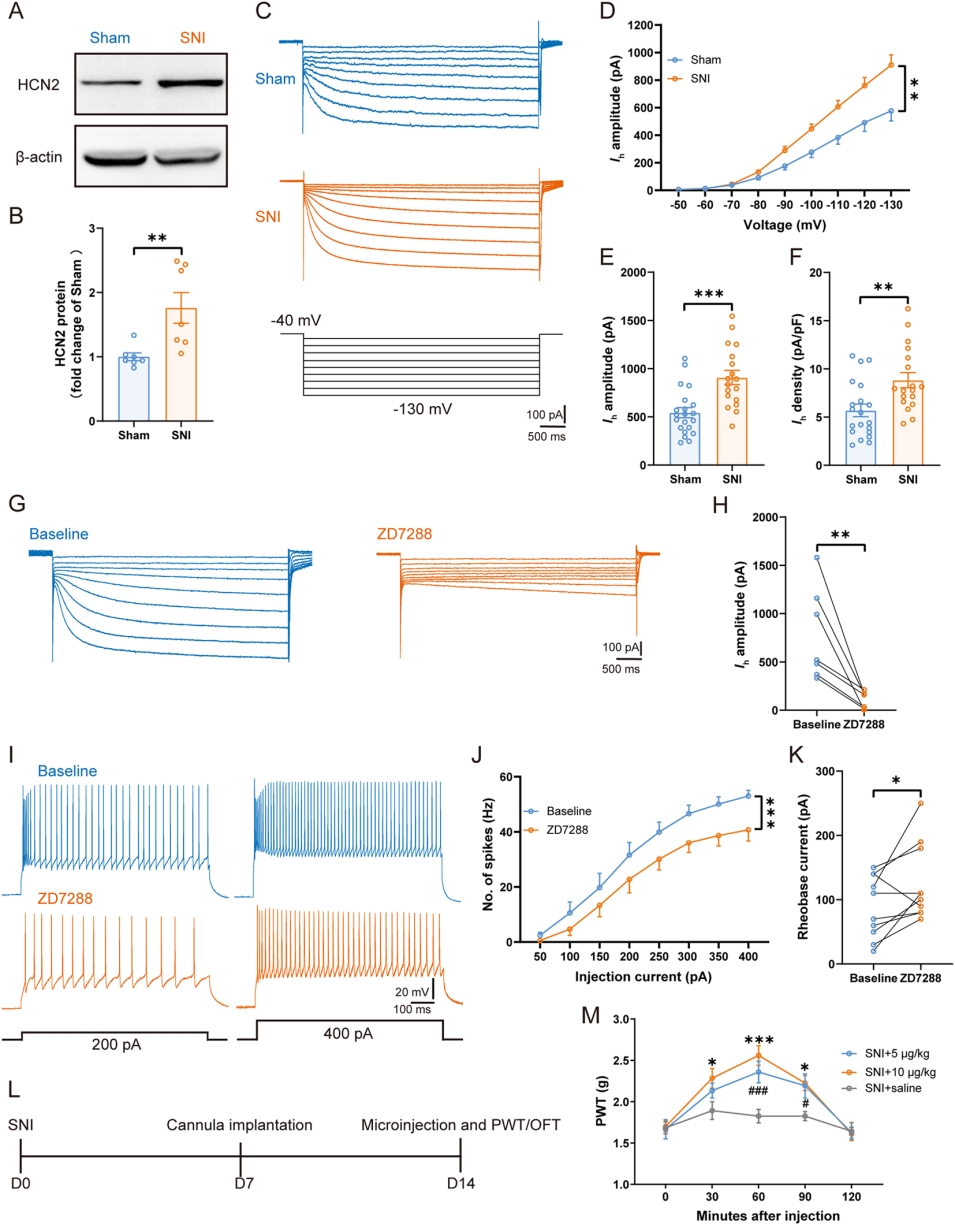
SNI increases the expression of HCN2 and *I*_h_ amplitude, and ZD7288 modulates TC neuronal activity and controls neuropathic pain behavior. **A, B** Representative (**A**) and quantitative analysis (**B**) of Western blot of HCN2 from sham and SNI mice (*n* = 7 per group, *t-*test). **C** Representative traces of *I*_h_ in VPL TC neurons from sham and SNI mice. **D** *I*_h_ amplitude recorded at different voltages in VPL TC neurons in sham and SNI mice (*n* = 21 cells from 10 sham mice, *n* = 18 cells from 13 SNI mice, two-way ANOVA). **E, F** Averaged *I*_h_ amplitude (**E**) and density (**F**) recorded at − 130 mV from VPL TC neurons in sham and SNI mice (*n* = 21 cells from 10 sham mice, *n* = 18 cells from 13 SNI mice, *t-*test). **G** Representative traces of *I*_h_ at baseline and with perfusion of ZD7288. **H** Statistical data showing the *I*_h_ amplitude at baseline and with perfusion of ZD7288 (*n* = 7 cells from 3 mice, *t-*test). **I** Representative traces of spikes recorded from VPL TC neurons at baseline and with perfusion of ZD7288. **J** Frequency-current (*F*-*I*) curves showing the number of spikes of VPL TC neurons in response to a series of 1-s current pulses from 50 pA to 400 pA at 50-pA steps at baseline and with perfusion of ZD7288 (*n* = 11 cells from 4 mice, two-way ANOVA). **K** Rheobase current at baseline and with perfusion of ZD7288 (*n* = 9 cells from 3 mice, *t-*test). **L** Timeline of experimental procedures. **M** Effects of microinjection of saline and ZD7288 on PWT in SNI mice (*n* = 6 per group, *P <0.05, ***P <0.001: SNI + 10 μg/kg ZD7288 group *vs* SNI + saline group; ^#^*P* <0.05, ^###^*P* <0.001: SNI + 5 μg/kg ZD7288 group *vs* SNI + saline group, two-way ANOVA). *P <0.05, **P <0.01, ***P <0.001 for **B, D, E, F, H, J**, and **K**. VPL, ventral posterolateral thalamus; TC, thalamocortical; PWT, paw withdrawal threshold; HCN2, hyperpolarization-activated cyclic nucleotide-gated channel 2; D0, day 0

The critical role of the VPL–S1HL circuit during neuropathic pain raises the question of the possible molecular mechanisms involved in this circuit. HCN2 plays an important role in the development and maintenance of neuropathic pain [25, 30]. Further, HCN2 is the predominant subunit in TC neurons, and the spike frequency of TC neurons is decreased in *Hcn2*-knockout mice [37, 40, 41]. A previous study revealed that the immunoreactivity and expression of HCN1 and HCN2 in the VPL are increased in rats with chronic constriction injury [33]. Therefore, we focused on HCN1 and HCN2 and used Western blot analysis to assess the expression levels of HCN1 and HCN2 in the VPL at 14 days after SNI. Compared with the sham mice, the expression of HCN2 was increased in SNI mice [sham: 1.00 ± 0.06 *vs* SNI: 1.76 ± 0.24, *t*_(12)_ = 3.07, *P* <0.01] (Fig. 5A, B). However, there was no difference between the two groups in the expression of HCN1 [sham: 1.00 ± 0.14 *vs* SNI: 0.94 ± 0.19, *t*_(8)_ = 0.27, *P* = 0.79] (Fig. S4). We then applied the whole-cell patch-clamp to TC neurons in the VPL to directly record *I*_h_ by clamping the membrane voltage from − 50 to − 130 mV in 10-mV steps (Fig. 5C). In SNI mice, the *I*_h_ amplitude was markedly higher than in sham controls [*F*_(1, 23)_ = 9.85, *P* <0.01] (Fig. 5D), suggesting an enhanced function of HCN channels. At − 130 mV, the *I*_h_ amplitude [SNI: 908.40 ± 73.91 pA *vs* sham: 543.60 ± 52.59 pA, *t*_(37)_ = 4.10, *P* <0.001] (Fig. 5E) and *I*_h_ density [SNI: 8.82 ± 0.79 pA/pF *vs* sham: 5.69 ± 0.66 pA/pF, *t*_(36)_ = 3.08, *P* <0.01] (Fig. 5F) were dramatically increased in SNI TC neurons. However, we did not detect any difference between the two groups in the half-activation potential (*V*_0.5_) [SNI: − 85.18 ± 0.89 mV *vs* sham: − 85.30 ± 1.68 mV, *t*_(30)_ = 0.06, *P* = 0.95] in TC neurons (Fig. S5).

### Effects of HCN2 Channel Activity on TC Neuronal Excitability and Neuropathic Pain Behavior

To explore whether the increased expression and function of the HCN2 channel is obligatory for the hyperactivity of TC neurons as well as nociceptive behavior, we suppressed the HCN2 channel *via* pharmacological or virus knockdown techniques. Bath application of ZD7288 (10 μmol/L) markedly decreased the *I*_h_ amplitude of TC neurons [Baseline: 776.60 ± 179.40 pA *vs* ZD7288: 109.70 ± 33.27 pA measured at − 130 mV, *t*_(6)_ = 3.89, *P* <0.01] (Fig. 5G, H). Moreover, ZD7288 reduced the number of depolarizing current-elicited spikes [*F*_(1, 11)_ = 20.37, *P* <0.001] (Fig. 5I, J) and increased the rheobase current compared to baseline [Baseline: 94.55 ± 14.98 pA *vs* ZD7288: 130.90 ± 17.81 pA, *t*_(10)_ = 2.58, *P* <0.05] (Fig. 5K), suggesting the involvement of HCN channels in regulating TC neuronal excitability. Next, we compared the mechanical threshold after acute microinjection of ZD7288 into the VPL of SNI mice (Fig. 5L). The positions of the cannulae were verified post-mortem by immunofluorescence staining (Fig. S6A). Notably, in the OFT, we did not find any modulation on the locomotor activity by ZD7288 as no difference was found in total ambulation [*F*_(2, 15)_ = 1.25, *P* = 0.29] (Fig. S6B), suggesting that injection of ZD7288 into the VPL does not affect locomotor activity. However, compared to the saline groups, ZD7288 significantly alleviated the mechanical allodynia in SNI mice at 60 (*P* <0.001) and 90 (*P* <0.05) min after injection at a dose of 5 μg/kg, and at 30 (*P* <0.05), 60 (*P* <0.001), and 90 (*P* <0.05) min at a higher dose (10 μg/kg) (Fig. 5M). Thus, these results demonstrate that the pharmacological blockade of HCN2 decreases TC neuronal activity and neuropathic pain behavior.

**Fig. 6:**
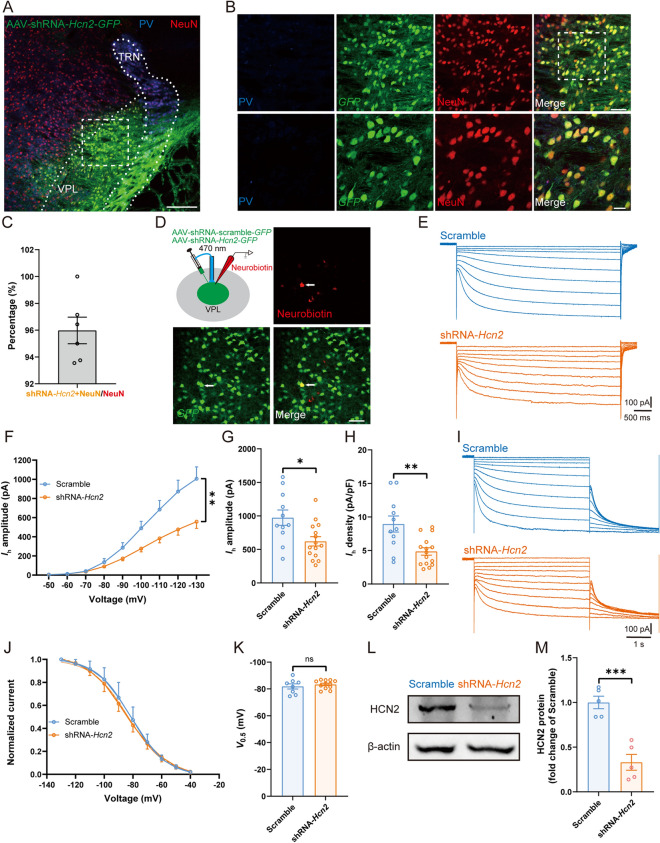
Virus knockdown of HCN2 decreases the *I*_h_ amplitude and expression. **A, B** Representative (**A**) and magnified (**B**) images of AAV-shRNA*-Hcn2-GFP* injection in the VPL. Scale bars, **A**: 200 μm; **B**: upper, 50 μm; lower, 20 μm. **C** Percentage of shRNA-*Hcn2*-positive neurons co-labeled with NeuN in the VPL. **D** Schematic of AAV-shRNA-*Hcn2-* or *scrambled-GFP* injection into the VPL and whole-cell recording in brain slices. Neurobiotin was used to confirm the recorded GFP-positive neuron. Scale bar, 50 μm. **E** Representative traces of *I*_h_ in VPL TC neurons from scrambled and shRNA-*Hcn2* mice. **F** *I*_h_ amplitude recorded at different voltages from VPL TC neurons in scrambled and shRNA-*Hcn2* mice (*n* = 11 cells from 5 scrambled mice, *n* = 15 cells from 6 shRNA-*Hcn2* mice, two-way ANOVA). **G, H** Averaged *I*_h_ amplitude (**G**) and density (**H**) at − 130 mV of VPL TC neurons in scrambled and shRNA-*Hcn2* mice (*n* = 11 cells from 5 scrambled mice, *n* = 15 cells from 6 shRNA-*Hcn2* mice, *t-*test). **I** Representative traces of *I*_h_ activation in VPL TC neurons in scrambled and shRNA-*Hcn2* mice. **J** Normalization of the voltage-activation determined from tail currents in scrambled and shRNA-*Hcn2* mice fitted with the Boltzmann equation (*n* = 8 cells from 5 scrambled mice, *n* = 12 cells from 6 shRNA-*Hcn2* mice). **K** *V*_0.5_ of *I*_h_ activation in scrambled and shRNA-*Hcn2* mice (*n* = 8 cells from 5 scrambled mice, *n* = 12 cells from 6 shRNA-*Hcn2* mice, *t-*test). **L, M** Representative (**L**) and quantitative analysis (**M**) of Western blots of HCN2 from scrambled and shRNA-*Hcn2* mice (*n* = 5 mice per group, *t-*test). For **F–H, K**, and **M**: *P <0.05, **P <0.01, ***P <0.001; ns, no significant difference. VPL, ventral posterolateral thalamus; TC, thalamocortical; PV, parvalbumin; TRN, thalamic reticular nucleus; HCN2, hyperpolarization-activated cyclic nucleotide-gated channel 2

To investigate the effect of HCN2 knockdown on pain behavior and TC neuronal excitability, we specifically knocked down HCN2 expression in the VPL by the AAV-shRNA-*Hcn2*-*GFP* vector. GFP marked infected neurons. Three weeks after injection, immunofluorescence staining showed a broad distribution of GFP-labeled neurons in the VPL (Fig. 6A, B), reaching a transduction efficiency of 95.98% ± 0.99% of VPL neurons, as indicated by confocal co-labeling of GFP with the neuronal marker NeuN (Fig. 6C). In an attempt to determine the efficiency of HCN2 knockdown, we made *in vitro* electrophysiological recordings from GFP-positive neurons distinguished by 470-nm LED light. Neurobiotin was loaded into a subset of these neurons during recording, and the co-localization of neurobiotin with GFP was further verified (Fig. 6D). As expected, compared with neurons expressing scrambled control, shRNA-*Hcn2* greatly reduced the *I*_h_ amplitude [*F*_(1, 18)_ = 9.54, *P* <0.01] (Fig. 6E, F). At − 130 mV, the *I*_h_ amplitude [shRNA-*Hcn2*: 620.50 ± 69.52 pA *vs* scrambled: 973.9 ± 114.80 pA, *t*_(24)_ = 2.78, *P* <0.05] (Fig. 6G) along with the *I*_h_ density [shRNA-*Hcn2*: 4.85 ± 0.55 pA/pF *vs* scrambled: 8.95 ± 1.19 pA/pF, *t*_(24)_ = 3.41, *P* <0.01] (Fig. 6H) were dramatically smaller in HCN2 knockdown neurons than in controls. No significant difference was found in *V*_0.5_ between shRNA-*Hcn2* and scrambled groups [shRNA-*Hcn2*: − 83.36 ± 0.92 mV *vs* scrambled: − 81.87 ± 1.94 mV, *t*_(18)_ = 0.77, *P* = 0.45] (Fig. 6I–K). In parallel, Western blots confirmed that shRNA-*Hcn2* greatly decreased the expression of HCN2 compared to the scrambled control [shRNA-*Hcn2*: 0.33 ± 0.09 *vs* scrambled: 1.00 ± 0.07, *t*_(8)_ = 5.92, *P* <0.001] (Fig. 6L, M).

**Fig. 7:**
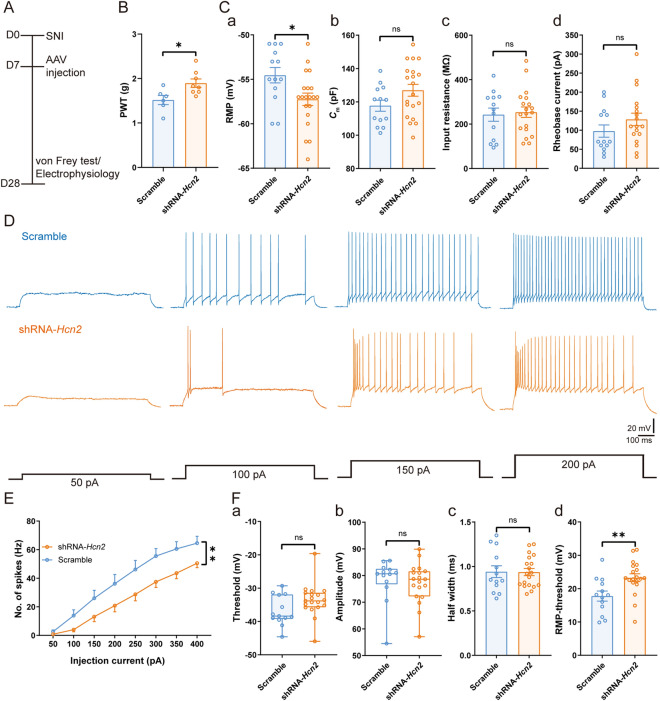
Virus knockdown of HCN2 decreases the excitability of TC neurons and mechanical allodynia in SNI. **A** Timeline of experimental procedures. **B** PWT in scrambled (*n* = 6) and shRNA-*Hcn2* (*n* = 8) mice, *t-*test. **C** Summary of the passive membrane properties of VPL TC neurons from scrambled and shRNA-*Hcn2* mice. **Ca** RMP; **Cb** *C*_m_; **Cc** input resistance; **Cd** rheobase current (*n* = 13 cells from 5 scrambled mice, *n* = 19 cells from 6 shRNA-*Hcn2* mice, *t-*test). **D** Representative traces of spikes recorded from VPL TC neurons from scrambled and shRNA-*Hcn2* mice. **E** Frequency-current (*F*-*I*) curves showing the number of spikes in VPL TC neuronal responses to a series of 1-s current pulses from 50 pA to 400 pA in 50-pA steps from scrambled and shRNA-*Hcn2* mice (*n* = 13 cells from 5 scrambled mice, *n* = 19 cells from 6 shRNA-*Hcn2* mice, two-way ANOVA). **F** Summary of the active membrane properties in sham and SNI mice. **Fa** Spike threshold; **Fb** Spike amplitude; **Fc** Spike half-width; **Fd** RMP-spike threshold (*n* = 13 cells from 5 scrambled mice, *n* = 19 cells from 6 shRNA-*Hcn2* mice, Mann-Whitney *U* test for **Fa** and **Fb**, *t-*test for **Fc** and **Fd**). For **B, C, E**, and **F**: *P <0.05, **P <0.01, ***P <0.001; ns, no significant difference. VPL, ventral posterolateral thalamus; TC, thalamocortical; PWT, paw withdrawal threshold; SNI, spared nerve injury; HCN2, hyperpolarization-activated cyclic nucleotide-gated channel 2; D0, day 0; RMP, resting membrane potential; *C*_m_, membrane capacitance

Notably, mechanical allodynia was significantly alleviated in SNI mice transfected with shRNA-*Hcn2* [shRNA-*Hcn2*: 1.89 ± 0.09 g *vs* scrambled: 1.52 ± 0.10 g, *t*_(12)_ = 2.68, *P* <0.05] (Fig. 7A, B). In addition, compared with the scrambled control, the RMP was more hyperpolarized in HCN2-knockdown neurons [shRNA-*Hcn2*: − 57.25 ± 0.69 mV *vs* scrambled: − 54.54 ± 0.86 mV, *t*_(31)_ = 2.44, *P* <0.05] (Fig. 7Ca). No significant difference was found between the two groups in *C*_m_ [shRNA-*Hcn2*: 126.90 ± 3.54 pF *vs* scrambled: 117.70 ± 3.30 pF, *t*_(31)_ = 1.80, *P* = 0.08] (Fig. 7Cb), input resistance [shRNA-*Hcn2*: 253.70 ± 23.55 MΩ *vs* scrambled: 242.50 ± 29.62 MΩ, *t*_(30)_ = 0.30, *P* = 0.77] (Fig. 7Cc), and rheobase current [shRNA-*Hcn2*: 128.30 ± 16.35 pA *vs* scrambled: 97.69 ± 16.14 pA, *t*_(29)_ = 1.30, *P* = 0.21] (Fig. 7Cd). Of note, we found a significant decrease in spiking frequency in shRNA-*Hcn2* compared with the scrambled group [*F*_(1, 19)_ = 10.47, *P* <0.01] (Fig. 7D, E). Spike threshold (shRNA-*Hcn2*: − 33.62 ± 1.13 mV *vs* scrambled: − 36.77 ± 1.24 mV, *P* = 0.08, Mann-Whitney test) (Fig. 7Fa), spike amplitude (shRNA-*Hcn2*: 77.14 ± 1.73 mV *vs* scrambled: 78.31 ± 2.27 mV, *P* = 0.38, Mann-Whitney test) (Fig. 7Fb), and spike half-width [shRNA-*Hcn2*: 0.93 ± 0.04 ms *vs* scrambled: 0.94 ± 0.07 ms, *t*_(30)_ = 0.06, *P* = 0.95] (Fig. 7Fc) remained unchanged. However, HCN2 knockdown raised the potential difference between spike threshold and RMP [shRNA-*Hcn2*: 23.27 ± 1.17 mV *vs* scrambled: 17.77 ± 1.51 mV, *t*_(30)_ = 2.91, *P* <0.01] (Fig. 7Fd). Overall, these data confirmed that maladaptive HCN2 function in VPL TC neurons is required for SNI-induced nociceptive hypersensitivity. Furthermore, we knocked down HCN2 in the VPL TC neurons projecting to the S1HL (VPL^S1HL^ TC neurons) using cre-Loxp techniques. As in Figs. 6 and 7, knocking down HCN2 in the VPL^S1HL^ TC neurons also reduced the *I*_h_ amplitude and the excitability of VPL^S1HL^ TC neurons and relieved the SNI-induced nociceptive hypersensitivity (Fig. S7).

### VPL HCN2 Channels Regulate Synaptic Transmission in the VPL–S1HL Circuit in Neuropathic Pain

**Fig. 8:**
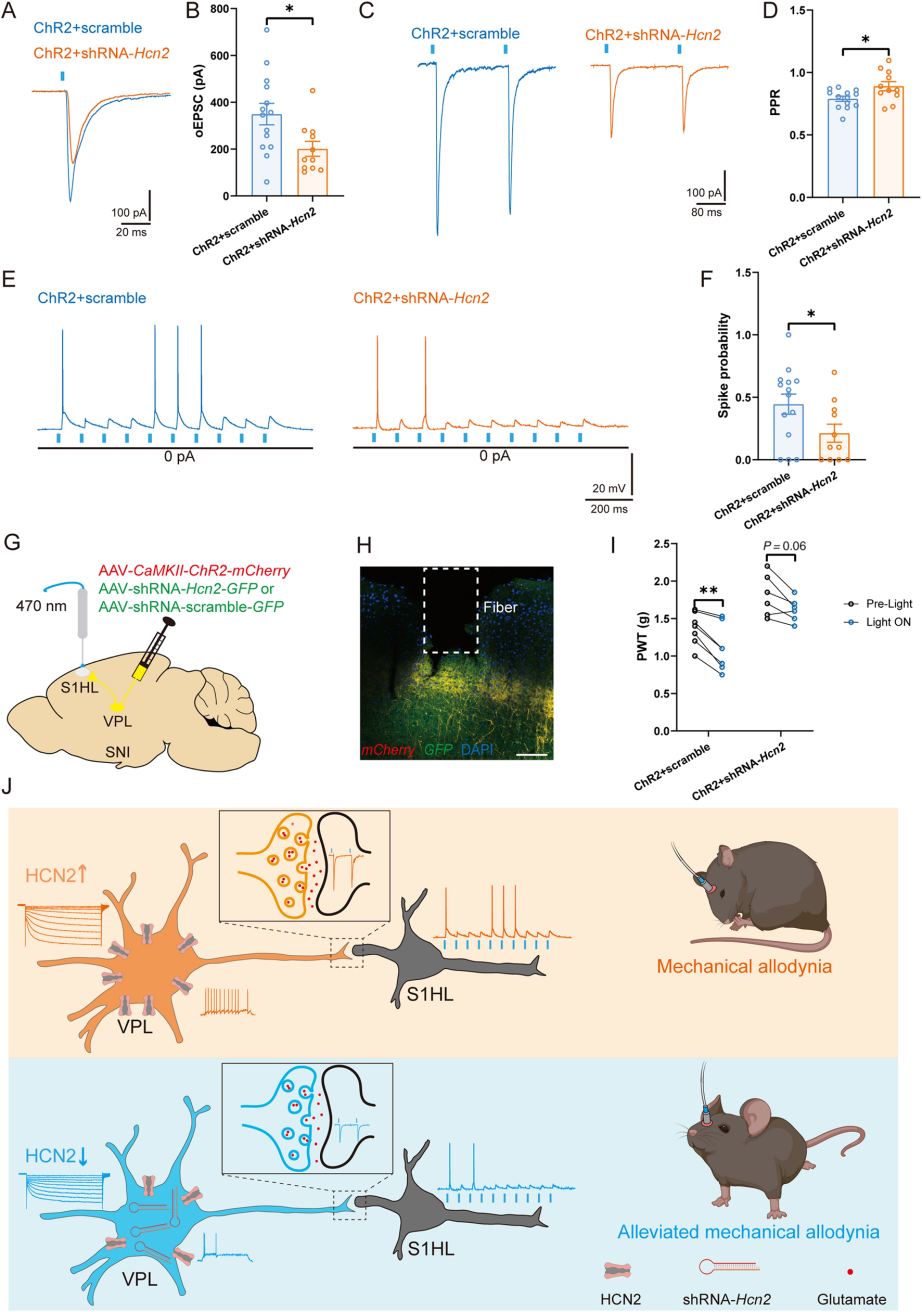
VPL HCN2 channels regulate the synaptic transmission of the VPL–S1HL circuit. **A, B** Representative traces (**A**) and statistical data (**B**) of oEPSC amplitude recorded from S1HL L4 RS neurons in ChR2 + scrambled and ChR2 + shRNA-*Hcn2* mice (*n* = 14 cells from 6 ChR2 + scrambled mice, *n* = 11 cells from 4 ChR2 + shRNA-*Hcn2* mice, *t-*test). **C, D** Representative traces (**C**) and statistical data (**D**) of PPR in S1HL L4 RS neurons in ChR2 + scrambled and ChR2 + shRNA-*Hcn2* mice (*n* = 13 cells from 6 ChR2 + scrambled mice, *n* = 11 cells from 4 ChR2 + shRNA-*Hcn2* mice, *t-*test). **E, F** Representative traces (**E**) and statistical data (**F**) of spikes elicited by VPL axon terminals in S1HL L4 RS neurons in ChR2 + scrambled and ChR2 + shRNA-*Hcn2* mice (*n* = 14 cells from 6 ChR2 + scrambled mice, *n* = 11 cells from 4 ChR2 + shRNA-*Hcn2* mice, *t-*test). **G, H** Schematic (**G**) and representative confocal image (**H**) of AAV*-CaMKII-ChR2-mCherry* and AAV-shRNA*-HCN2-* or scrambled*-GFP* injection into the VPL and fiber implanted in the S1HL. Scale bar, 100 μm. **I** Effects of optogenetic activation of VPL axon terminals in the S1HL on PWT in ChR2 + scrambled and ChR2 + shRNA-*Hcn2* mice (*n* = 7 per group, *t-*test). **J** Working model: In neuropathic pain, increased expression of HCN2 in the VPL results in augmented TC neuronal excitability in the VPL and enhanced synaptic transmission in the VPL–S1HL circuit. Virus knockdown of HCN2 in the VPL decreases the excitability of VPL TC neurons, the ectopic synaptic transmission of the VPL–S1HL circuit, and SNI-induced hyperalgesia. For **B, D, F**, and **I**: *P <0.05, **P <0.01, ***P <0.001. VPL, ventral posterolateral thalamus; S1HL, hindlimb region of the primary somatosensory cortex; RS, regular spiking; TC, thalamocortical; PPR, paired-pulse ratio; PWT, paw withdrawal threshold; oEPSC, optogenetic excitatory postsynaptic current; SNI, spared nerve injury; HCN2, hyperpolarization-activated cyclic nucleotide-gated channel 2; L4, layer IV

Although HCN2 channels modulate TC neuronal excitability, it is unknown whether the enhanced function of HCN2 in SNI mice is sufficient to activate the VPL–S1HL circuit and lead to neuropathic pain. Therefore, we finally used a dual-virus approach to express ChR2 in both the soma and the axons of TC neurons and down-regulate the expression of HCN2 in the VPL (Fig. S8). HCN2 knockdown in the VPL dramatically decreased the amplitude of oEPSCs [ChR2 + shRNA-*Hcn2*: 201.30 ± 31.89 pA *vs* ChR2 + scrambled: 349.40 ± 45.79 pA, *t*_(23)_ = 2.51, *P* <0.05] (Fig. 8A, B) and increased the PPR [ChR2 + shRNA-*Hcn2*: 0.89 ± 0.03 *vs* ChR2 + scrambled: 0.79 ± 0.02, *t*_(22)_ = 2.46, *P* <0.05] (Fig. 8C, D) of RS neurons in L4 of the S1HL. Moreover, the probability of spikes elicited by optogenetic activation of VPL output to the S1HL was also decreased after HCN2 down-regulation [ChR2 + shRNA-*Hcn2*: 0.21 ± 0.07 *vs* ChR2 + scrambled: 0.45 ± 0.08, *t*_(23)_ = 2.08, *P* <0.05] (Fig. 8E, F). Notably, optogenetic activation of the VPL–S1HL circuit in the ChR2 + scrambled group significantly alleviated the SNI-induced mechanical hypersensitivity [Pre-light: 1.36 ± 0.08 g *vs* Light ON: 1.10 ± 0.11 g, *t*_(6)_ = 5.46, *P* <0.01]. However, HCN2 knockdown partly reversed the optogenetic stimulation-induced nociceptive responses [Pre-light: 1.77 ± 0.10 g *vs* Light ON: 1.61 ± 0.05 g, *t*_(6)_ = 2.35, *P* = 0.06] (Fig. 8G–I).

In summary, we identified an HCN2 channel activity-dependent increase in excitation of the VPL-to-S1HL projection is sufficient to induce nociceptive hypersensitivity.

## Discussion

A previous study implicated the VPL HCN channel in neuropathic pain [33]. In this study, we demonstrated that the dysfunction of HCN2 in the VPL activates the TC neurons and VPL–S1HL circuit to elicit neuropathic pain by using *in vitro* patch-clamp recording, *in vivo*, and *in vitro* optogenetic techniques. These results further indicate that VPL HCN2 channels may be a therapeutic target for neuropathic pain.

The VPL has been identified as a well-validated brain region responsible for integrating nociceptive information [17, 22, 42]. Besides, human brain imaging studies have shown that mean VPL volume is strongly associated with average and worst pain intensity, and abnormal connectivity between the VPL and other brain regions, such as the postcentral gyrus and insula, has been confirmed in patients suffering from pain [15, 16, 43]. In rodents, the spontaneous spike rate and the oscillation of local field potentials in the VPL have been reported to be increased in remifentanil-induced hyperalgesia [21]. In line with these findings, we further revealed an increase in VPL TC neuronal activity in the SNI-evoked neuropathic pain state. Importantly, our experiments demonstrated that optogenetic activation or inhibition of TC neurons is sufficient to worsen or alleviate the mechanical hypersensitivity in SNI mice. However, a previous study [14] demonstrated that neurons in the posterior thalamic nucleus but not the VPL are activated 7 days after SNI. The differences in our results raise the possibility that changes in TC neuronal excitability in the VPL may be time-dependent following peripheral nerve injury. Besides, we found that optogenetic activation of the VPL TC neurons induced mechanical allodynia, with an absence of response to optogenetic inhibition in sham mice. These results suggest that TC neuronal excitability may be relatively low in the physiological state, thus further suppressing the activity of TC neurons does not affect pain perception. Taken together, we demonstrated that the potentiated TC neuronal activity is an indispensable factor for driving pathological pain.

Previous studies have reported that the S1HL receives projections from TC neurons [14, 44, 45], and the firing pattern of VPL neurons is linked to pain-induced cortical synchronization and ectopic TC connections [22, 46]. Thus, it is possible to assume that the functional synaptic connectivity from the VPL to the S1HL may be altered by peripheral neuropathy. In line with the previous studies [14, 47, 48], we confirmed a monosynaptic excitatory connection between VPL TC neurons and S1HL L4 RS neurons. Moreover, our data showed that SNI increased the mEPSC frequency, firing frequency, oEPSC amplitude, and spike probability, and reduced the PPR of S1HL L4 RS neurons. Of note, we found that optogenetically inhibiting or activating VPL TC neuronal output to the S1HL *in vivo* yielded decreased or increased pain behavior, similar to our results of direct modulation of TC somatic excitability in SNI mice. This is consistent with a previous study demonstrating that migraine causes activity-dependent synaptic plasticity as well as excitatory-inhibitory imbalance in S1 [44]. However, we found that optical stimulation of VPL–S1HL axon terminals was insufficient to produce mechanical hypersensitivity in sham mice. This might be brought about by a weaker ChR2-driven excitatory effect on TC neuronal axon terminals than on their cell bodies. Together, all these findings demonstrate that changes in the TC circuit are essential for pathological pain hypersensitivity.

Among the four subunits of HCN channels, HCN2 is abundantly expressed in the VPL [49, 50] and has been identified as an indispensable substrate for regulating TC neuronal activity, as HCN2 deletion leads to a reduction in spike numbers in response to varied depolarizing currents [37, 51]. However, whether the malfunction of HCN2 leads to changes in TC neuronal activity and the VPL–S1HL circuit was unknown. Unlike prior research [33], we demonstrated that SNI markedly augmented the *I*_h_ amplitude of TC neurons and up-regulated HCN2 but not HCN1 expression in the VPL. Reasons for this discrepancy in the changes in HCN1 and HCN2 expression may be attributable to the different species and models we used in our studies. Furthermore, in line with a previous study that showed that genetic deficiency in, or pharmacological antagonism of, HCN2 causes a decrease in DRG firing and nociceptive hypersensitivity [30], we found that TC neuronal hyperexcitability was reduced by pharmacological blockade or virus knockdown of HCN2. Our *in vivo* behavioral test also showed that microinjection of ZD7288 into the VPL alleviated the mechanical allodynia without causing sedation in SNI mice, which was duplicated by shRNA-*Hcn2* injection. Notably, we found that HCN2 knockdown in the VPL decreased oEPSC amplitude and increased PPR in the VPL–S1HL circuit in pathological pain states, suggesting that HCN2 might dysregulate VPL outputs of the VPL–S1HL circuit in the SNI by a presynaptic mechanism. These findings suggest that increased TC neuron firing and VPL–S1HL circuit activity are specifically due to the enhanced activity of HCN2 channels. To the best of our knowledge, this is the first study to examine the effect of HCN2 on the dysregulation of VPL outputs of the VPL–S1HL circuit in persistent pain states.

The normal functions of HCN channels are finely regulated by the neurochemical environment, including small intracellular molecules, protein kinases, and extracellular neurotransmitters [52]. We have not determined here why the expression and function of HCN2 in TC neurons are up-regulated following peripheral nerve injury. Several underlying mechanisms are possible, such as the enhanced activity of p38 mitogen-activated protein kinase [53] and calmodulin-dependent protein kinase II [54]. However, the detailed mechanisms need to be further investigated.

In conclusion, the present study demonstrates that HCN2 plays a crucial role in the TC neuronal hyperexcitability, which in turn, activates VPL–S1HL excitatory synaptic transmission and promotes neuropathic pain behavior after peripheral nerve injury (Fig. 8J).

## Supplementary Information

Below is the link to the electronic supplementary material.

Supplementary file1 (PDF 2043 kb)
