# Supplementary material for: Thalamocortical Circuit Controls Neuropathic Pain via Up-regulation of HCN2 in the Ventral Posterolateral Thalamus
Source: Neurosci Bull. 2022 Dec 20;39(5):774–92. doi: 10.1007/s12264-022-00989-5 (PMC10169982; doi:10.1007/s12264-022-00989-5)
Supplement: Supplementary file 1 — Supplementary file1 (PDF 2043 kb) [file 12264_2022_989_MOESM1_ESM.pdf]

Supplementary Information

Supplementary Figures

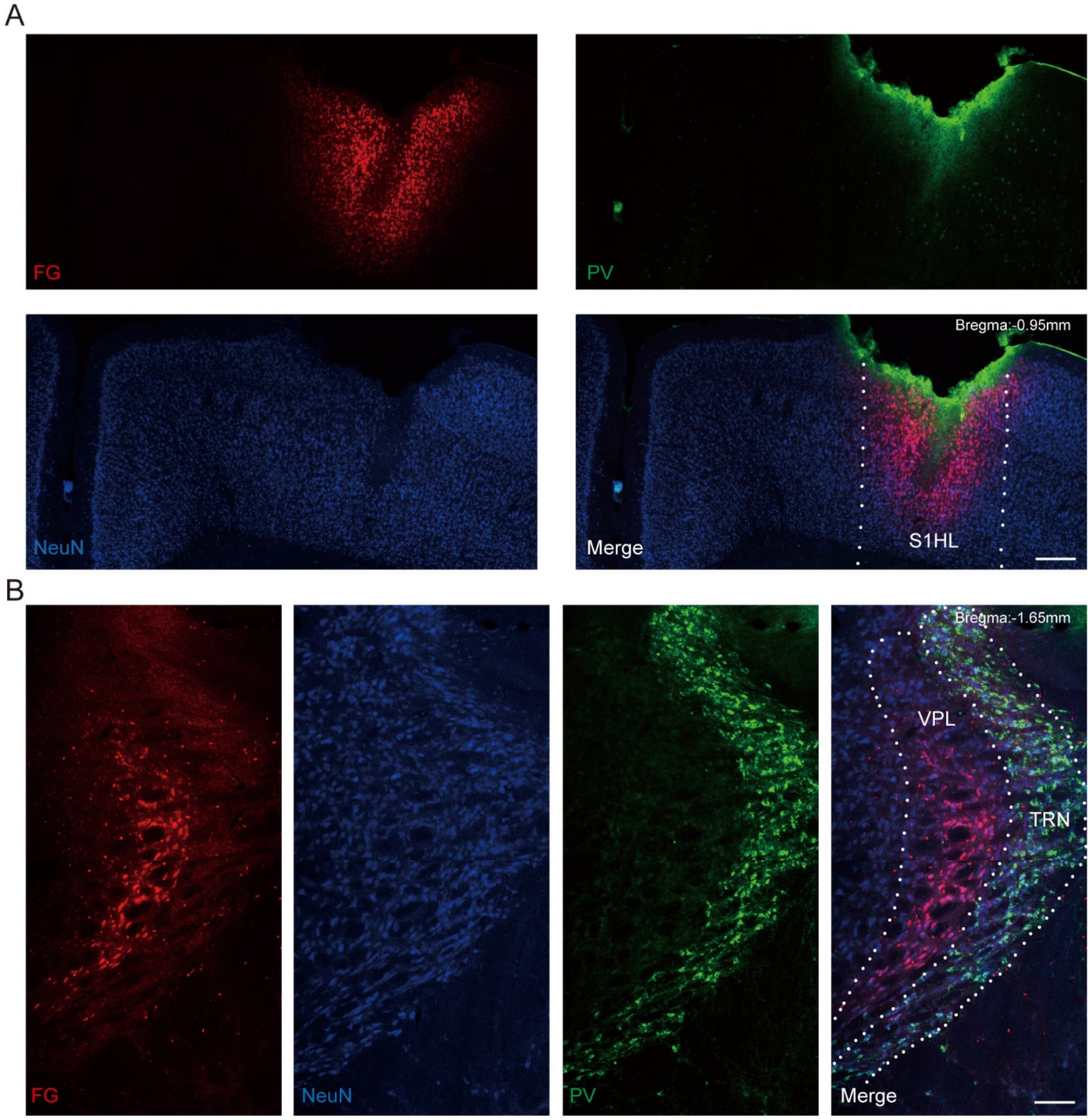

Fig. S1 Retrograde tracer FG identifies the VPL–S1HL circuit. **A** Representative images of retrograde tracer FG injection in the S1HL. Scale bar, 200 μm. **B** Representative images of retrograde tracer FG-labeled cell bodies in the VPL. Scale bar, 100 μm. VPL, ventral posterolateral thalamus; S1HL, hindlimb region of the primary somatosensory cortex; PV, parvalbumin; TRN, thalamic reticular nucleus; FG, Fluoro-Gold.

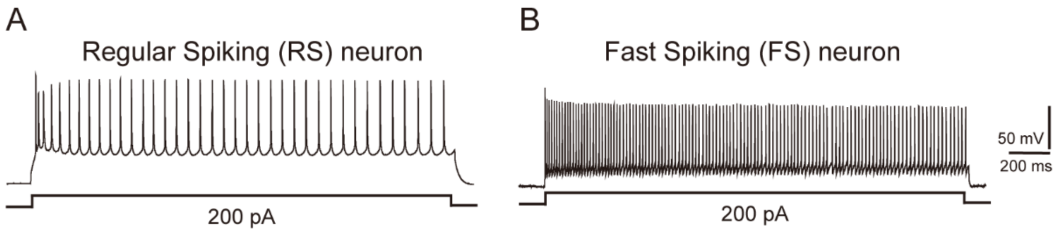

Fig. S2 Representative traces of the firing patterns of S1HL neurons. **A** A regular spiking neuron. **B** A fast spiking neuron.

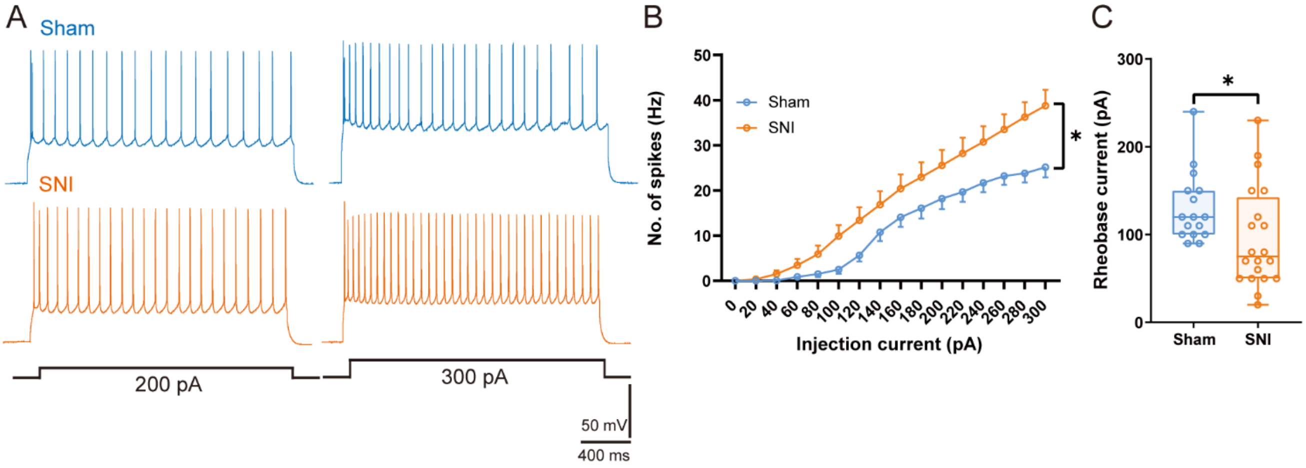

Fig. S3 S1HL layer IV (L4) RS neuronal excitability is altered in neuropathic pain states. **A** Representative traces of spikes recorded from S1HL L4 RS neurons in sham and SNI mice. **B** Frequency-current (*F*-*I*) curves showing the number of spikes of S1HL L4 RS neuronal responses to a series of 1-s current pulses from 0 pA to 300 pA in 20-pA steps in sham and SNI mice (*n* = 16 cells from 7 sham mice, *n* = 17 cells from 8 SNI mice, two-way ANOVA). **C** Statistical data showing the rheobase current of S1HL L4 RS neurons in sham and SNI mice (*n* = 16 cells from 7 sham mice, *n* = 20 cells from 8 SNI mice, Mann-Whitney *U* test). **P* <0.05. SNI, spared nerve injury; S1HL, hindlimb region of the primary somatosensory cortex; RS, regular spiking.

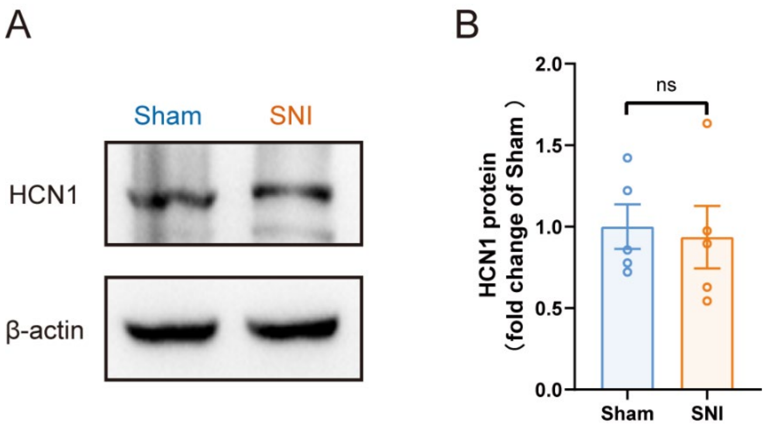

Fig. S4 No changes in HCN1 of the VPL in neuropathic pain. **A** Representative blots and **B** quantitative analysis of Western blots of HCN1 from sham and SNI mice (*n* = 5 per group, *t*-test; ns, no significant difference). VPL, ventral posterolateral thalamus; SNI, spared nerve injury; HCN1, hyperpolarization-activated cyclic nucleotide-gated channel 1.

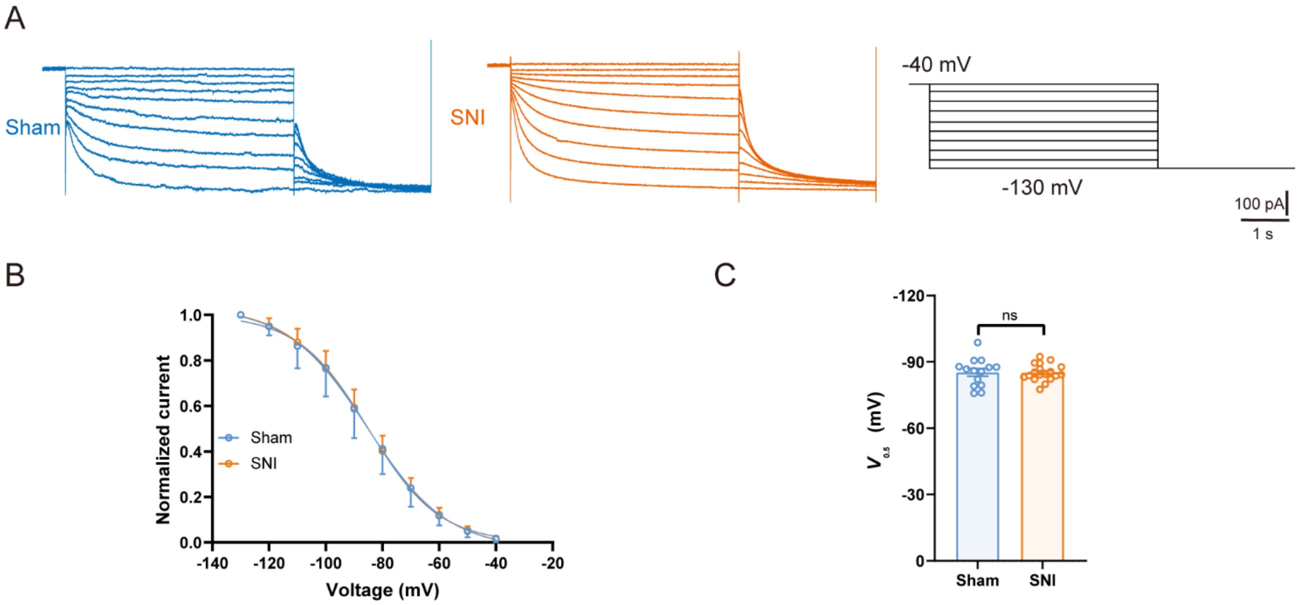

Fig. S5 No changes in I_h activation of VPL TC neurons in neuropathic pain. **A** Representative traces of I_h activation in VPL TC neurons. **B** Normalization of the voltage-activation determined from tail currents in sham and SNI mice fitted with the Boltzmann equation (*n* = 14 cells from 10 sham mice, *n* = 18 cells from 11 SNI mice). **C** $V_{0.5}$ of I_h activation from sham and SNI mice (*n* = 14 cells from 10 sham mice, *n* = 18 cells from 11

SNI mice, *t*-test). ns, no significant difference. VPL, ventral posterolateral thalamus; SNI, spared nerve injury; TC, thalamocortical.

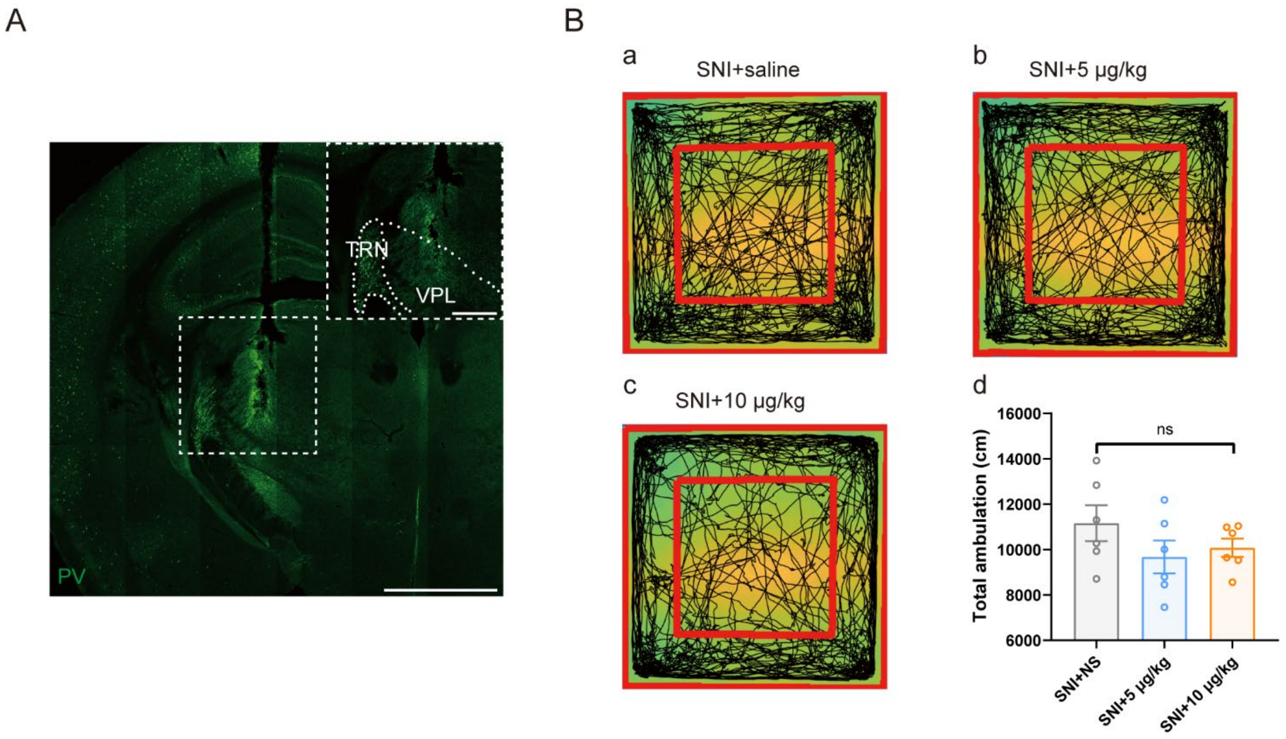

Fig. S6 No changes in locomotor activity with ZD7288 injection. **A** Representative confocal image of cannula implantation in the VPL. Scale bars, 1 mm and 400 μm (inset). **B** Representative image of the OFT. **Ba** SNI + saline; **Bb** SNI + 5 μg/kg ZD7288; **Bc** SNI + 10 μg/kg ZD7288; **Bd** Statistical data showing the total ambulation of mice in the OFT (n = 6 for each group, one-way ANOVA; ns, no significant difference). OFT, open field test; SNI, spared nerve injury.

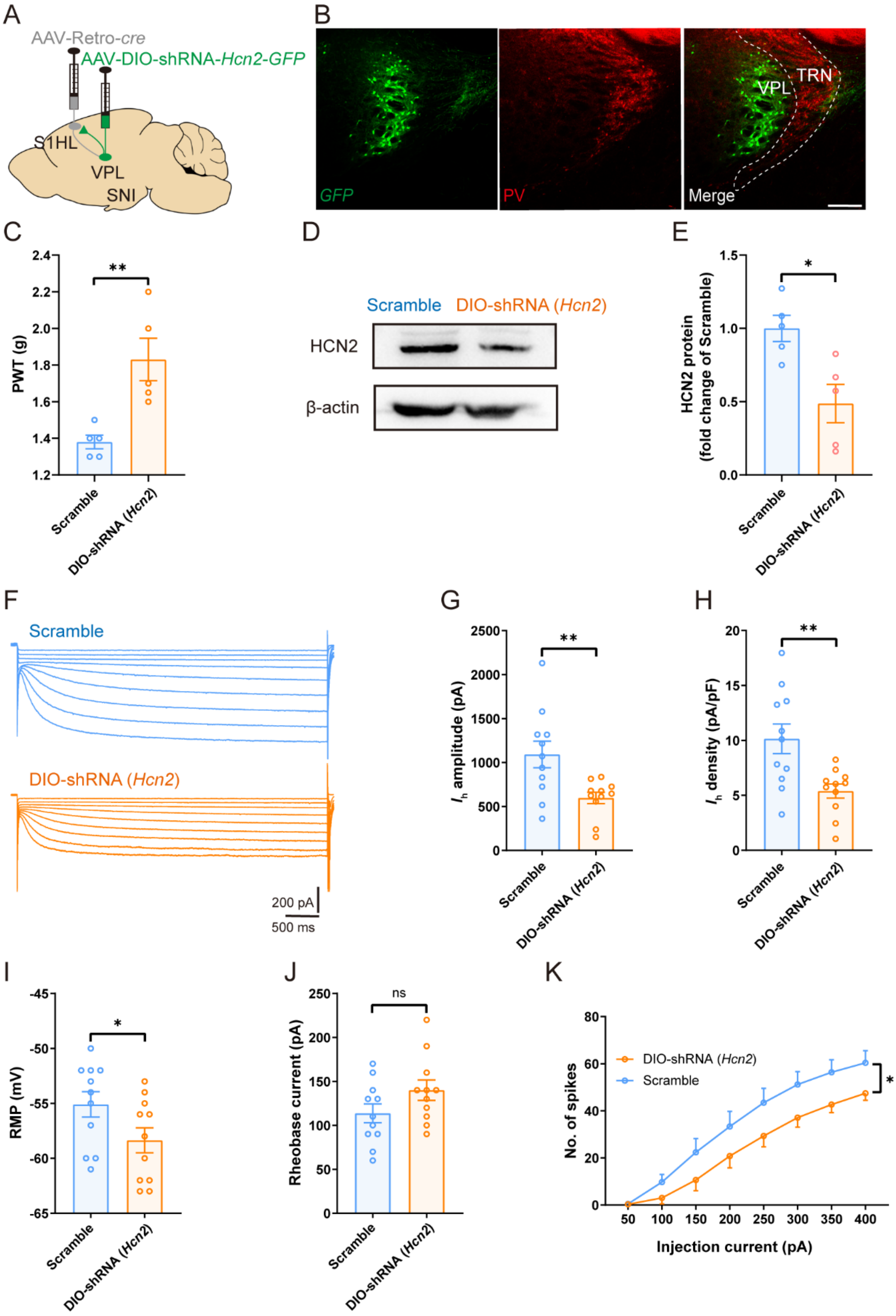

Fig. S7 Virus knockdown of HCN2 in VPL^{S1HL} TC neurons in SNI mice. **A** Schematic of AAV-Retro-cre in the S1HL and AAV-DIO-shRNA-*Hcn2-GFP* in the VPL. **B** Representative confocal images of AAV-DIO-shRNA-*Hcn2-GFP* in the VPL. Scale bar, 200 μm. **C** PWT in the scrambled and DIO-shRNA

(*Hcn2*) mice [scrambled: 1.38 ± 0.04 g *vs* DIO-shRNA (*Hcn2*): 1.83 ± 0.11 g, $t_{(8)}$= 3.70, P <0.01, n = 5 per group, *t*-test]. **D, E** Representative blots (**D**) and quantitative analysis (**E**) of Western blots of HCN2 from scrambled and DIO-shRNA (*Hcn2*) mice [scrambled: 1.00 ± 0.08 *vs* DIO-shRNA (*Hcn2*): 0.49 ± 0.13, $t_{(8)}$= 3.25, P <0.05, n = 5 per group, *t*-test]. **F** Representative traces of I_h in VPL^{S1HL} TC neurons. **G, H** Averaged I_h amplitude [scrambled: 1091.00 ± 151.50 pA *vs* DIO-shRNA (*Hcn2*): 596.00 ± 64.94 pA, $t_{(20)}$= 3.00, P <0.01, *t*-test] (**G**) and density [scrambled: 10.14 ± 1.35 pA/pF *vs* DIO-shRNA (*Hcn2*): 5.39 ± 0.64 pA/pF, $t_{(20)}$= 3.18, P <0.01, *t*-test] (**H**) at −130 mV of VPL^{S1HL} TC neurons in scrambled and DIO-shRNA (*Hcn2*) mice [n = 11 cells from 4 scrambled mice, n = 11 cells from 4 DIO-shRNA (*Hcn2*) mice, *t*-test]. **I** Summary of the RMP of VPL^{S1HL} TC neurons in scrambled and DIO-shRNA (*Hcn2*) mice [scrambled: −55.09 ± 1.16 mV *vs* DIO-shRNA (*Hcn2*): −58.36 ± 1.14 mV, $t_{(20)}$= 2.11, P <0.05, n = 11 cells from 4 scrambled mice, n = 11 cells from 4 DIO-shRNA (*Hcn2*) mice, *t*-test]. **J** Summary of the rheobase current of VPL^{S1HL} TC neurons in scrambled and DIO-shRNA (*Hcn2*) mice [scrambled: 113.60 ± 10.73 pA *vs* DIO-shRNA (*Hcn2*): 140.00 ± 11.60 pA, $t_{(20)}$= 1.67, P = 0.11, n = 11 cells from 4 scrambled mice, n = 11 cells from 4 DIO-shRNA (*Hcn2*) mice, *t*-test]. **K** Frequency-current (*F-I*) curves showing the numbers of spikes in VPL^{S1HL} TC neuronal responses to a series of 1-s current pulses from 50 pA to 400 pA in 50-pA steps [$F_{(7, 54)}$= 2.55, P <0.05, n = 11 cells from 4 scrambled mice, n = 11 cells from 4 DIO-shRNA (*Hcn2*) mice, two-way ANOVA]. *P <0.05, **P <0.01, ns, no significant difference. VPL, ventral posterolateral thalamus; PV, parvalbumin; TRN, thalamic reticular nucleus; HCN2, hyperpolarization-activated cyclic nucleotide-gated channel 2; S1HL, hindlimb region of the primary somatosensory cortex; PWT, paw withdrawal threshold; SNI, spared nerve injury; RMP, resting membrane potential.

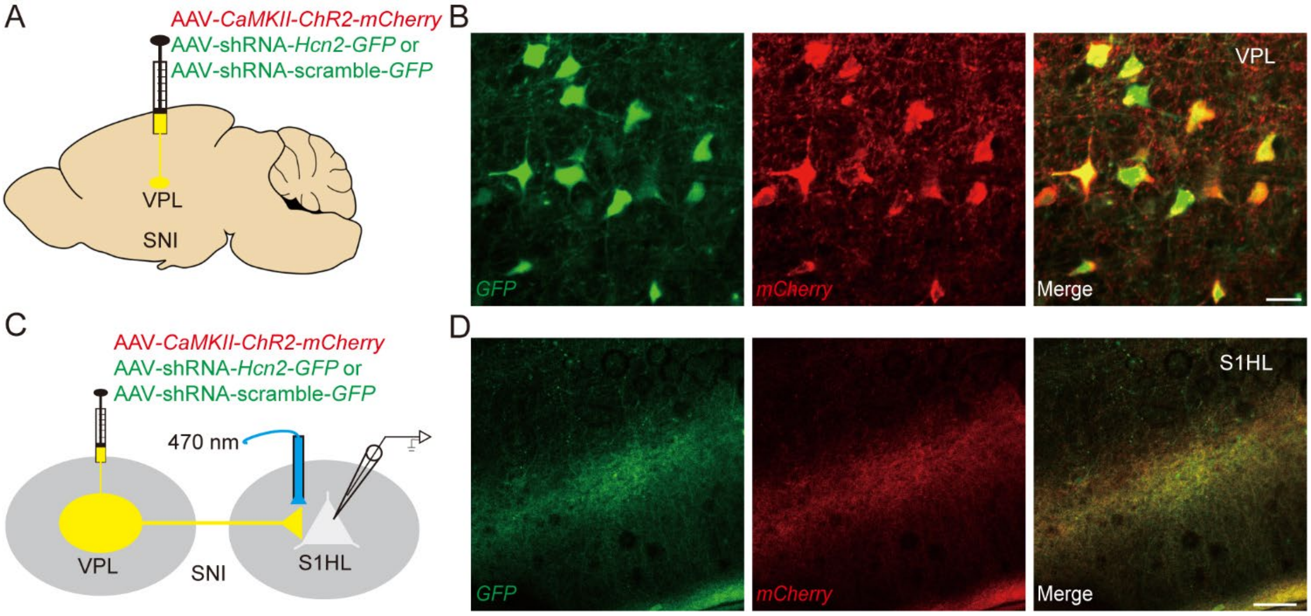

Fig. S8 AAV-*CaMKII-ChR2-mCherry* and AAV-shRNA-*Hcn2*- or scrambled-*GFP* injection into the VPL. **A** Schematic of AAV-*CaMKII-ChR2-mCherry* and AAV-shRNA-*Hcn2*- or scrambled-*GFP* injection into the VPL. **B** Representative confocal images of *AAV-CaMKII-ChR2-mCherry* and AAV-shRNA-*Hcn2*- or scrambled-*GFP* injection into the VPL. Scale bar, 20 µm. **C** Schematic of AAV-*CaMKII-ChR2-mCherry* and AAV-shRNA-*Hcn2*- or scrambled-*GFP* injection into the VPL and whole-cell recording in the S1HL. **D** Representative confocal images of AAV-*CaMKII-ChR2-mCherry* and AAV-shRNA-*Hcn2*- or scrambled-*GFP*-positive axon terminal expression in the S1HL. Scale bar, 100 µm. VPL, ventral posterolateral thalamus; S1HL, hindlimb region of the primary somatosensory cortex; SNI, spared nerve injury.
